# Genetic characterization of a core collection of flax (*Linum usitatissimum* L.) suitable for association mapping studies and evidence of divergent selection between fiber and linseed types

**DOI:** 10.1186/1471-2229-13-78

**Published:** 2013-05-06

**Authors:** Braulio J Soto-Cerda, Axel Diederichsen, Raja Ragupathy, Sylvie Cloutier

**Affiliations:** 1 Department of Plant Science, University of Manitoba, 66 Dafoe Road, Winnipeg, MB R3T 2N2, Canada; 2 Cereal Research Centre, Agriculture and Agri-Food Canada, 195 Dafoe Rd, Winnipeg, MB R3T 2M9, Canada; 3 Plant Gene Resources of Canada, Agriculture and Agri-Food Canada, 107 Science Place, Saskatchewan, SK S7N 0X2, Canada; 4 Present address: Agriaquaculture Nutritional Genomic Center, CGNA, Genomics and Bioinformatics Unito, Km 10 Camino Cajón-Vilcún, INIA Temuco, Chile

**Keywords:** Genetic diversity, Population structure, Linkage disequilibrium, Association mapping, *Linum usitatissimum*, Fiber flax, Linseed, Divergent selection

## Abstract

**Background:**

Flax is valued for its fiber, seed oil and nutraceuticals. Recently, the fiber industry has invested in the development of products made from linseed stems, making it a dual purpose crop. Simultaneous targeting of genomic regions controlling stem fiber and seed quality traits could enable the development of dual purpose cultivars. However, the genetic diversity, population structure and linkage disequilibrium (LD) patterns necessary for association mapping (AM) have not yet been assessed in flax because genomic resources have only recently been developed. We characterized 407 globally distributed flax accessions using 448 microsatellite markers. The data was analyzed to assess the suitability of this core collection for AM. Genomic scans to identify candidate genes selected during the divergent breeding process of fiber flax and linseed were conducted using the whole genome shotgun sequence of flax.

**Results:**

Combined genetic structure analysis assigned all accessions to two major groups with six sub-groups. Population differentiation was weak between the major groups (F_ST_ = 0.094) and for most of the pairwise comparisons among sub-groups. The molecular coancestry analysis indicated weak relatedness (mean = 0.287) for most individual pairs. Abundant genetic diversity was observed in the total panel (5.32 alleles per locus), and some sub-groups showed a high proportion of private alleles. The average genome-wide LD (*r*^2^) was 0.036, with a relatively fast decay of 1.5 cM. Genomic scans between fiber flax and linseed identified candidate genes involved in cell-wall biogenesis/modification, xylem identity and fatty acid biosynthesis congruent with genes previously identified in flax and other plant species.

**Conclusions:**

Based on the abundant genetic diversity, weak population structure and relatedness and relatively fast LD decay, we concluded that this core collection is suitable for AM studies targeting multiple agronomic and quality traits aiming at the improvement of flax as a true dual purpose crop. Our genomic scans provide the first insights into candidate regions affected by divergent selection in flax. In combination with AM, genomic scans have the ability to increase the power to detect loci influencing complex traits.

## Background

Flax (*Linum usitatissimum* L.) is an annual, self-pollinated species with a genome size of ~ 370 Mb [1]. The species is believed to have originated in either the Middle East or Indian regions [2] and spread throughout Asia and Europe, prior to its introduction into the New World [3]. Divergent selection applied over thousands of years has resulted in fiber and linseed types which are the same species but differ considerably in morphology, anatomy, physiology and agronomic performance [4]. Fiber flax cultivars are taller and less branched and are grown in the cool-temperate regions of China, the Russian Federation and Western Europe [3]. Linseed cultivars are shorter, more branched, larger seeded and are grown over a wider area in continental climate regions of Canada, India, China, the United States and Argentina [3]. Flax provides raw materials for food, medicine and textiles and, as such, it has been of great importance to human culture and development for more than 8,000 years [5]. Linseed oil is well-known for its health benefits mainly attributed to its high content of omega-3 alpha linolenic acid (55-57%). Linseed oil has been used for centuries in paints and varnishes because of its unique drying properties attributable to its distinctive fatty acid composition [6]. Consumption of ground seeds adds nutritional benefits because flax seeds are also a rich source of lignans, compounds that have anticancer properties [7]. In the last decade, the fiber industry has devoted some effort to develop high-value products from linseed stems with applications in the pulp, technical fiber and biofuel industries [4,8]. Therefore, understanding the genetic diversity of flax collections is important for the continued improvement of this crop as well as for its development into a truly dual purpose crop [8].

Initial diversity assessments in flax were carried out using morphological parameters [9-12] and isozymes [13,14]. In recent years, molecular marker systems such as randomly amplified polymorphic DNA (RAPD), amplified fragment length polymorphism (AFLP), inter-simple sequence repeat (ISSR), simple sequence repeat (SSR) and inter-retrotransposon amplified polymorphism (IRAP) have been used to measure genetic variation and relationships in cultivars and landraces of flax [15-29]. However, most of these previous studies assessed either few marker loci or few genotypes.

World gene banks store approximately 48,000 accessions of flax germplasm [30]. In Canada, a world collection of approximately 3,500 accessions of cultivated flax is maintained by Plant Gene Resources of Canada (PGRC). This collection has traditionally been deployed in flax breeding through a variety of conventional strategies [3]. In 2009, the Total Utilization Flax Genomics (TUFGEN; http://www.tufgen.ca) project was initiated in Canada to generate genomics resources for flax and to apply them to an array of traits for the ultimate purpose of flax improvement. The TUFGEN project has developed numerous genomics resources including molecular markers [23,29,31], genetic maps [32,33], a physical map and bacterial artificial chromosome end sequences [1], expressed sequence tags [34] and whole genome shotgun sequence [35]. To take advantage of these tools, a core collection of 407 flax accessions capturing the breadth of the phenotypic diversity of the PGRC collection was assembled.

Quantitative trait loci (QTL) and association mapping (AM) are complementary approaches for the identification of marker-trait association. The first utilizes biparental mapping populations to monitor the co-segregation of QTL and marker loci. The second utilizes germplasm collections to identify QTL-marker correlations based on LD [36]. QTL analysis has limited mapping resolution due to the accumulation of few meiosis events in a single cross, but it is not affected by population structure which can be a source of spurious association in AM. Conversely, AM can achieve higher mapping resolution through high numbers of historical recombination events in germplasm collections. An ideal association panel should harbor the broadest genetic diversity because this is often correlated with a rapid LD decay necessary to resolve complex trait variation(s) to a single gene or nucleotide [37]. Null or weak population structure and a low level of relatedness among individuals of the germplasm collection are also desirable. Thus, genetic diversity, population structure, familial relatedness and LD patterns need to be assessed prior to AM analyses to fully exploit their advantages for flax genetic improvement.

In this study we genotyped 407 flax accessions using 448 microsatellite loci. The overall goal was to evaluate the usefulness of this flax core collection for AM studies. Our specific goals were: (1) to investigate the genetic diversity; (2) to estimate the levels of population structure and assess familial relatedness; (3) to detect the patterns of LD; and (4) to identify non-neutral genomic regions potentially underlying divergent selection between fiber and linseed types.

## Results

### Phylogenetic analysis

**Figure 1:**
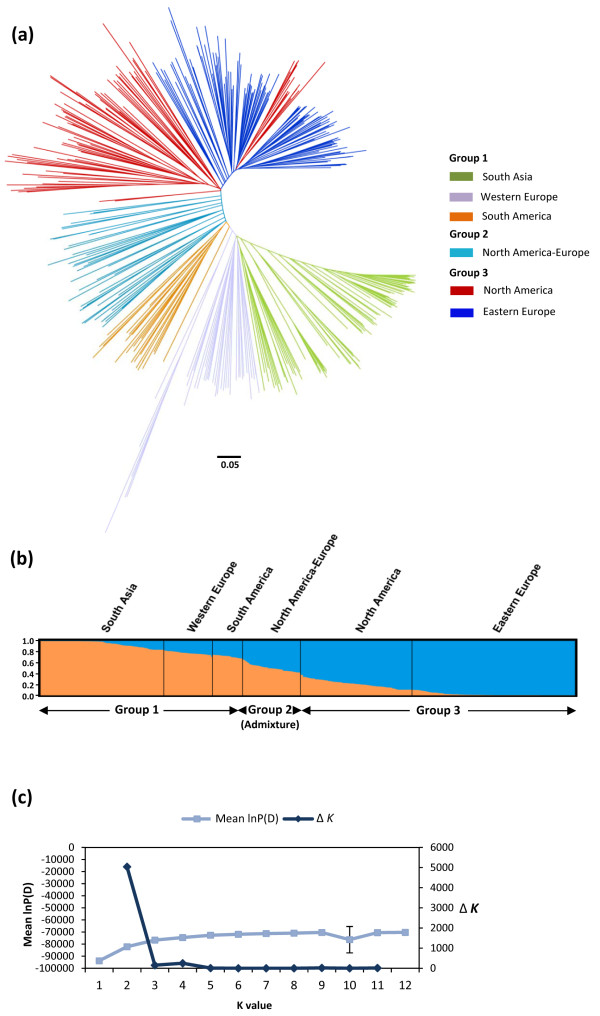
**Genetic relationships and population structure of the 407 flax accessions of the core collection. (a)** Phylogenetic tree created using the Neighbor-joining (NJ) algorithm [62] and information from 414 neutral SSRs. Colored clusters represent the sub-groups within major groups. The scale bar indicates the Nei [62] minimum genetic distance. **(b)** Bayesian clustering (STRUCTURE *K* = 2). Sub-groups within groups are distributed according to the clustering obtained by the NJ analysis. Accessions with a membership coefficient Q < 0.7 were classified as admixture Group 2. **(c)** Average log-likelihood values (mean lnP(D) ± SD for 10 iterations) and ad-hoc statistic Δ*K*[70] for *K* values ranging from 1 to 12.

Based on 414 neutral loci, the phylogenetic analysis using the NJ algorithm partitioned the 407 accessions into two major groups and one admixed group (Figure 1a; Additional file 1: Table S1). Group 1 (G1) was composed of 155 accessions that were further subdivided into three sub-groups representing accessions from South Asia, Western Europe and South America. The South Asian sub-group included mostly accessions from India, Pakistan and Afghanistan while the Western European sub-group contained mostly accessions from France, Portugal and Germany but also from Romania and Turkey. The South American sub-group included accessions from Argentina and Uruguay. Group 3 (G3) had 206 accessions distributed into two sub-groups, namely North America and Eastern Europe. The North American sub-group clustered cultivars and breeding materials originating exclusively from Canada and the U.S.A. However, not all North American accessions clustered within that sub-group. A few of these accessions were included in the Eastern European sub-group which otherwise included mostly accessions from Russia, Ukraine, Romania, Poland and Lithuania. This sub-group included 90% of the fiber flax accessions present in the core collection. Within the Eastern European sub-group, the geographic origin and industrial use overlapped, including fiber flax accessions from the Netherlands, the former Soviet Union and the U.S.A. The admixed group (G2), namely the North American/European group, had 43 accessions from the U.S.A., Canada and European countries.

### Population structure

A total of 259 loci meeting the neutrality criteria, with LD < 0.4 and distributed across the 15 linkage groups, were included in these analyses. Similar to the phylogenetic analysis based on the NJ algorithm, the PCoA revealed the presence of two major groups albeit with some admixture among sub-groups (Additional file 2: Figure S1a). Coordinate 1 and 2 explained 65.8% of the total genetic variation. The Bayesian-based clustering approach implemented in STRUCTURE also identified two groups according to the Δ*k* approach (Figure 1b, c). Based on the estimated membership coefficient (*Q*), the South Asian, Western European and South American sub-groups (*Q* > 0.70) could be clustered together within G1, and the North American and Eastern European sub-groups (*Q* > 0.70) could be similarly clustered within G3. The North American/European sub-group (G2) was mostly an admixture of the other two major groups. Taken together, the NJ, PCoA and STRUCTURE analyses all agreed with respect to the distribution of the 407 flax accessions into two major groups. Additionally, the NJ and STRUCTURE analyses agreed in partitioning the collection into six sub-groups, with few differences among sub-groups. The high proportion of shared alleles revealed by the PCoA and STRUCTURE analyses was confirmed by the weak population structure as measured by the coefficient of population differentiation (F_ST_ = 0.094, P < 0.001) between G1 and G3. The level of differentiation between sub-groups ranged from 0.02 (P < 0.001, North America *vs* Eastern Europe) to 0.16 (P < 0.001, Eastern Europe *vs* South Asia) (Additional file 2: Figure S1b).

### Molecular coancestry

**Figure 2:**
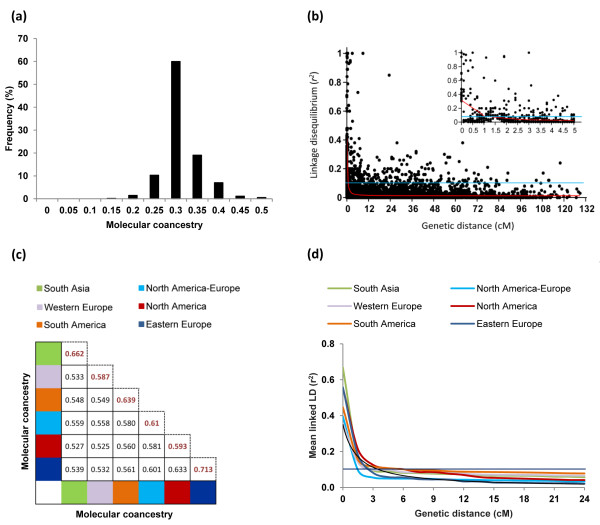
**Distribution of pairwise molecular coancestry estimates and linkage disequilibrium decay. (a)** Global pairwise molecular coancestry estimates of the 407 flax accessions of the core collection. Only kinship values ranging from 0 to 0.5 are shown. **(b)** Scatter plot of LD decay (*r*^2^) against the genetic distances (cM) for pairs of linked SSRs across the 15 linkage groups. The inner panel shows a detailed view of LD decay for markers located within 5 cM. The decay curves were plotted according to Breseghello and Sorells [75]. The blue line represents the threshold level of significance (*r*^2^ = 0.1). The red line represents the average genome-wide LD of linked markers. **(c)** Pairwise molecular coancestry estimates [72] within each of the six sub-groups. The diagonal values correspond to the intra sub-group molecular coancestry. **(d)** Average genome-wide LD decay curves for linked markers within each of the six sub-groups.

Based on the alleles of the 448 microsatellites, the average molecular coancestry between any two flax accessions was 0.287 in the core collection as a whole. Approximately 70% of the pairwise coancenstry estimates ranged from 0.1 to 0.3 (Figure 2a). The intra sub-group molecular coancestry ranged from 0.587 (Western Europe) to 0.713 (Eastern Europe). The pairwise molecular coancestry estimates ranged from 0.525 (North America *vs* Western Europe) to 0.633 (North America *vs* Eastern Europe) (Figure 2c). Overall, more than 80% of the pairwise molecular coancestry estimates in the core collection and sub-groups ranged from 0.114 to 0.350 and 0.525 to 0.601, respectively. The coancestry analysis indicated that most flax accessions had weak and moderate familial relatedness with the other accessions in the core collection and sub-groups respectively, which may be a reflection of the broad genetic diversity of the PGRC collection and the careful selection of accessions exercised while constructing the core collection.

### Genetic diversity

In the core collection, the 414 neutral microsatellites retained detected 2202 alleles (N_a_) (mean = 5.32/locus), out of which 1187 (54%) had a MAF < 0.05 and were considered rare alleles (R_a_). The total unbiased gene diversity (UH_e_) and the observed heterozygosity (H_o_) were 0.427 and 0.023, respectively. Allelic richness (R_s_) was estimated at 5.68, the inbreeding coefficient (F_IS_) at 0.946 and the PIC value at 0.374. The genetic diversity parameters were also estimated for the major groups and sub-groups (Table 1). The parameters N_a_, R_s_, ∏, R_a_ and PIC in G1 were superior to those in G3, even though the population size of G1 was 25% smaller than G3. The parameters H_o_ and F_IS_ across the core collection, the major groups and sub-groups are consistent with the predominantly self-pollinated nature of the species.

**Table 1:** **Genetic diversity parameters of the core collection for the two major groups** (**G1 and G3**), **the admixed group** (**G2**) **and their sub**-**groups**

**Population**	**N**^**1**^	**UH**_**e**_^**2**^	**H**_**o**_^**3**^	**N**_**a**_^**4**^	**R**_**s**_^**5**^	**∏**^**6**^	**R**_**a**_^**7**^	**F**_**IS**_^**8**^	**Polymorphic loci (%)**	**PIC**^**9**^
Core collection	407	0.427	0.023	2202	5.68	-	1187	0.946	100	0.374
Group 1	153	0.418	0.023	1978	4.37	547	925	0.944	99.8	0.361
South Asia	92	0.348	0.020	1510	2.85	116	542	0.931	95.9	0.305
Western Europe	37	0.448	0.017	1608	3.44	246	418	0.961	97.1	0.393
South America	24	0.395	0.047	1135	2.70	27	186	0.878	91.3	0.332
Group 2										
North Amer./Eur.	43	0.411	0.023	1341	2.91	32	324	0.933	96.4	0.352
Group 3	211	0.356	0.022	1613	3.44	183	683	0.933	99.1	0.332
North America	95	0.378	0.028	1362	2.69	73	424	0.932	98.6	0.334
Eastern Europe	116	0.300	0.020	1487	2.55	45	642	0.927	95.7	0.265

^1^ Number of accessions.

^2^ Unbiased gene diversity.

^3^ Observed heterozygosity.

^4^ Number of alleles.

^5^ Allelic richness and ^6^ number of private alleles estimated on a sample of balanced size using the rarefaction method [66].

^7^ Rare alleles < 5%.

^8^ Inbreeding coefficient.

^9^ Polymorphism information content.

**Table 2:** **Linkage disequilibrium in the core collection for the two major groups** (**G1 and G3**), **the admixed group** (**G2**) **and their six sub**-**groups**

**Population**	**Mean linked LD (*r***^***2***^**)**	**Mean unlinked LD (*r***^***2***^**)**	**95**^**th **^**percentile unlinked LD**	**Loci pairs in significant LD (%)**
Core collection	0.036	0.023	0.09	10.81
Group 1	0.047	0.035	0.14	8.10
South Asia	0.070	0.056	0.22	8.82
Western Europe	0.072	0.067	0.26	6.08
South America	0.084	0.067	0.25	8.75
Group 2				
North Amer./Eur.	0.040	0.032	0.12	8.08
Group 3	0.037	0.019	0.08	12.22
North America	0.061	0.030	0.11	15.68
Eastern Europe	0.036	0.020	0.08	10.86

### Linkage disequilibrium

To analyze LD variation, genetic distances for 293 microsatellites were available from the consensus linkage map of flax [33]. The average genome-wide distance between adjacent markers was 5.3 ± 2.4 cM. In the core collection, the average *r*^2^ values for linked and unlinked markers were 0.036 and 0.023, respectively. The 95^th^ percentile of *r*^2^ distribution for unlinked markers was 0.09 and 10.81% of the loci pairs were in significant LD. The average genome-wide LD decayed to 0.1 within 1.5 cM (Figure 2b). LD values within sub-groups and major groups are presented in Table 2. The average *r*^2^ values for linked and unlinked markers were higher in G1 than in G3 and the percentage of loci in significant LD was lower with 8.10% in G1 versus 12.22% in G3. Slower LD decay was observed within sub-groups (Figure 2d), ranging from 1.5 cM (North America-Europe) to 6.0 cM (South America), which could be attributed to the limited population size and narrow genetic diversity of some sub-groups as compared to the core collection. Regardless of the data set, i.e., core collection or inferred groups, the average *r*^2^ for linked markers remained higher than for unlinked markers, supporting physical linkage as the main determinant of LD in this core collection. The relatively rapid LD decay within the core collection suggested that high marker saturation will be required for effective AM. The slower LD decay within some of the sub-groups could be exploited for exploratory AM or coarse mapping.

### Identification of non-neutral loci

The fiber and linseed groups made of the 92 fiber accessions of the core collection and a random subset of 92 linseed accessions were subjected to bottleneck analysis [38]. The mode-shift test showed the typical L-shaped distribution of allele frequencies in both groups (data not shown), expected at mutation drift-equilibrium when rare alleles are numerous, thus suggesting absence of a recent bottleneck [39]. The sign test, however, indicated a heterozygosity excess (bottleneck) in the fiber group (P < 0.01) but not in the linseed group (P = 0.346). The population structure analysis showed a sharp peak of Δ*k* at *K* = 2 largely corresponding to the fiber and linseed types (Additional file 3: Figure S2a, b). Thus, no hierarchical population structure was detected and the two original groups (fiber and linseed) were adopted for posterior analyses. Distortion from neutral expectations was detected at 41, 13, 14 and 26 loci with EW, ln RH, LOSITAN and Arlequin, respectively (data not shown). A total of 9 loci (mean F_ST_ = 0.16) distributed across 7 linkage groups were significant in at least two of the four tests and were considered true outliers (Table 3). LD between these and their adjacent loci ranged from 0 to 0.10 in the fiber group and from 0 to 1.0 in the linseed group.

**Table 3:** Outlier analysis for divergent selection between fiber and linseed types

		**Outlier analysis**				**Highest LD**^**1**^		
**Locus**	**Linkage group**	**Ewens-Watterson**	**Ln RH**	**LOSITAN**	**Hierarchical**	**Fiber**	**Oil**	**F**_**ST**_
c206-s208_Lu128	2	n.s.	n.s.	***	**	0.03 (6.2)	0.03 (6.2)	0.43
c475-s917_Lu2021a	2	*	**	***	n.s.	0.01 (1.5)	0.03 (8.3)	0.04
c16-s156_Lu373	3	**	*	n.s.	n.s.	0.10 (1.9)	0.22 (1.9)	0.16
c36-s291_Lu176	5	**	n.s.	n.s.	*	0.02 (3.3)	0.06 (3.3)	0.27
c108-s305_Lu595	8	**	**	***	n.s.	0.00 (0.0)	0.00 (0.0)	0.10
c441-s225_Lu3189	8	*	n.s.	n.s.	*	0.05 (2.7)	0.31 (2.7)	0.32
c175-s86_Lu2824	9	*	**	***	n.s.	0.01 (4.1)	0.02 (6.9)	0.02
c306-s98_Lu765Bb	12	**	**	***	n.s.	0.09 (1.3)	1.00 (1.3)	0.07
c226-s280_Lu637	15	**	*	***	n.s.	0.02 (3.7)	0.13 (3.7)	0.01

* P < 0.05, ** P < 0.01, *** false discovery rate (FDR) < 0.05, n.s. not statistically significant.

^1^ Highest LD between the candidate locus and one of the two adjacent loci. The genetic distance (cM) at which the highest LD extent was observed is indicated in brackets.

The physical to genetic distance between c306-s98_Lu765Bb and its closest locus c306-s98_Lu3063 was estimated at 364 kb/cM. Considering an LD of 1 between them and a genetic distance estimate of 1.3 cM, we investigated a physical interval of 474 kb. A total of 98 genes were predicted in this interval, of which 59 showed significant similarities with high quality annotations of the UniProtKB protein database (Additional file 4: Table S2). The physical to genetic distance between c16-s156_Lu373 and c16-s156_Lu139 was estimated at 178 kb/cM with a moderate LD (*r*^2^ = 0.22). Consequently, the hitchhiking effects may not extend across the total genetic distance of 1.9 cM. We estimated the LD decay in both flax type groups to calculate an average genetic distance at which LD was strong (Additional file 3: Figure S2c). LD decayed to 0.4 within ≈ 0.2 cM, equivalent to 36 kb in which only five highly similar genes were predicted. The number of predicted genes from different scaffolds with weak LD (*r*^2^ < 0.2) having significant similarities to annotated proteins ranged from 3 (c175-s86_Lu2824, c206-s208_Lu128 and c441-s225_Lu3189) to 5 (c36-s291_Lu176 and c108-s305_Lu595) (Additional file 4: Table S2).

GO annotations could be assigned to ~60% of the predicted genes which are expressed in flax based on EST and protein evidence. Mapping of predicted proteins from 86 candidate genes to the UniProtKB database yielded 1,035 GO annotations as a result of multiple associations of individual proteins with multiple functions, processes or components [40] (Additional file 4: Table S2). The top four GO categories for molecular function were ‘binding’ (21.9%), ‘catalytic activity’ (14.4%), ‘nucleotide binding’ (10.6%) and ‘hydrolase activity’ (10%) (Additional file 5: Figure S3a). Similarly, functional characterization of proteins associated with candidate genes at non-neutral loci indicated unknown broad ‘biological processes’ (19.1%), followed by ‘cellular processes’ (16%), ‘metabolic processes’ (11.1%) and ‘response to stress’ (7.6%) (Additional file 5: Figure S3b). The candidate gene products were localized to membrane (11.8%) and intracellular locations (9.2%) (Additional file 5: Figure S3c). Approximately 4.6% of the predicted proteins were localized to the cell wall. Key genes associated with cell-wall biogenesis/modification, xylem identity, auxin regulation and fatty acid biosynthesis were identified among our candidate genes potentially affected by divergent selection in flax (Additional file 4: Table S2).

## Discussion

To contribute to long term sustainability of flax production and diversification, the germplasm stored in PGRC has comprehensively been characterized for morphologic, phenologic and agronomic characteristics [22]. This valuable phenotypic information enabled the construction of a flax core collection of 407 accessions to further flax genetic studies and improvement. Here, we report on the genetic characterization of the core collection based on 448 microsatellite loci which represents one of the largest flax genetic studies published to date [14-16,18-21,23-26,28,29,41].

### Genetic relationships and population structure

Understanding the genetic relationships and structure of core collections is critical to control false positives in AM [37]. The NJ tree grouped the 407 flax accessions mainly but not exclusively according to geographical origin. The presence of accessions from countries out of the geographical clusters could be explained by the fact that the passport data may be occasionally weak where the donor country is considered the country of origin. As a consequence, the names of the sub-groups were assigned according to the geographic origin of the majority of the accessions within them.

The South Asian sub-group of G1 was the most genetically distinct. Fu [20] reported similar results in 2,727 flax accessions assessed with 149 RAPD markers. However, in his study, the Indian subcontinent and Central Asia were considered related groups rather than a unified cluster. Differences in the marker systems and extent of the genome coverage (414 mapped microsatellite vs. 149 RAPD markers) could explain the resolution differences between studies. The active exchanges of flax germplasm between France, Germany, the United Kingdom and Hungary provide support for the Western European grouping [42]. The genetic relationships among G1 accessions were also supported by a weak population differentiation among sub-groups (F_ST_ = 0.05 - 0.11, Additional file 2: Figures S1a, b). Within G3, the North American sub-group reflects historical germplasm exchange between the U.S.A. and Canada [18]. The Eastern European sub-group contained most of the fiber flax accessions from the Netherlands and the former Soviet Union but it also included linseed accessions that were not intermixed. They were separated by a small group of U.S.A. accessions clustered within this sub-group. The U.S.A. accessions were mostly fiber type. Similar results observed in the population structure analyses and the lowest F_ST_ (0.02) between sub-groups (Additional file 2: Figures S1a, b) could explain the interstitial presence of the U.S.A. accessions. The two major groups supported by our combined approach showed weak population subdivision in support of the breadth of the genetic diversity captured in this collection, making it ideal for AM [36].

### Molecular coancestry

Strong population structure, familial relatedness, or both, may be significant in a core collection and would negatively impact AM. Yu et al. [43] developed a mixed linear model (MLM) which incorporates the pairwise kinship (*K* matrix) to correct for relatedness. Spurious associations cannot be controlled completely by population structure (*Q* matrix) [37,43]. Models incorporating a *K* matrix are generally superior in controlling the rate of false positives while maintaining statistical power as compared to those using only a *Q* matrix [43].

In self-pollinated crops or inbred lines, coancestry estimates tend to be higher than in outcrossing species because the high hererozygosity reduces the probability that two alleles observed at a locus are identical by state [44]. In our core collection, approximately 80% of the pairwise coancestry estimates ranged from 0.1 to 0.3, indicating that most of the lines had weak relatedness (Figure 2a). We anticipate that with the weak population structure and relatedness of the core collection, a MLM correcting for *K* should provide sufficient statistical power to control most of the false positive associations in future AM studies [43].

### Genetic diversity

A suitable core collection for AM should encompass as much phenotypic and molecular diversity as can be reliably measured in a given environment [36,37]. An average of 5.32 alleles per locus over 414 microsatellites was observed in our core collection. This value is higher than the range previously reported (2.72 – 3.46) [28,41,45,46]. This allelic diversity even exceeded that of a diverse sample of *L*. *usitatissimum* L. subsp. *angustifolium* (Huds.) Thell., (wild progenitor) and *L*. *usitatissimum* L. subsp. *usitatissimum* (4.62) [26]. This high value may be the result of the number of genotypes analyzed (407), the choice of the germplasm, the number of microsatellite loci (414 neutral out of 448) and the microsatellite repeat type and length [29,47].

A higher number of private alleles were observed in G1 as compared to G3 (Table 1). The Western European sub-group was particularly rich in private alleles with 246. Novel genetic variations, not previously sampled or utilized in modern flax breeding programs, may be present in this sub-group, offering unique alleles for broadening the diversity of flax gene pools. This is contrary to previous studies that have reported generally low genetic diversity of flax germplasm [18,21,23,26-28]. Although 85% of the accessions of our core collection are cultivars and breeding materials, the collection possesses abundant genetic diversity, an advantageous attribute for dissecting the genetic basis of QTL for immediate application in flax breeding [36,48].

### Linkage disequilibrium

Low LD demands the use of dense marker sets resulting in tight linkage between markers and QTL, an advantageous criterion for breeding applications because the predictive ability of a marker will be robust through generations [36]. The average *r*^2^ of the entire core collection was 0.036 and the average genome-wide LD decayed within 1.5 cM (Figure 2b). In self-pollinated species where recombination is less effective than in outcrossing species LD declines more slowly [36]. Nonetheless, the germplasm that makes up the collection plays a key role in LD variation because the extent of LD is influenced by the level of genetic variation captured by the target population. For example, in wild barley (*Hordeum vulgare* ssp. *spontaneum*), despite its high rate of self-fertilization (~98%), LD decayed within 2 kb, a value similar to that observed in maize, an outcrossing species [49]. The low LD of this core collection dictates the need for higher marker saturation to provide superior mapping resolution and QTL detection power by AM [50] as compared to using biparental linkage maps. Alternatively, selection of sub-groups with low F_ST_ and higher but similar levels of LD would require a reduced number of individuals and markers for exploratory AM.

The percentage of loci pairs in significant LD was fairly similar in each sub-group except for the North American and Eastern European sub-groups which registered the highest values, possibly reflecting their more intensive artificial selection and narrow germplasm [18]. Although our core collection did not behave as an unstructured large population, our combined analyses of population structure showed that G1 and G3 were weakly differentiated, representing two ancestral populations that minimize differences in LD and potentially the amount of spurious associations (Figures 1a, b). Thus, the results of our LD characterization within diverse genetic groups offer the versatility to perform cost-effective AM studies in flax by providing the fundamental characterization of the collection demonstrating its usefulness for AM.

### Identification of non-neutral loci

Flax is one of the few domesticated plants that have been subjected to disruptive selection [8]. North America almost exclusively grows linseed and, up until recently, the stems were considered more problematic than beneficial because of their slow field biodegradation. However, the use of short fibers has received increased attention in North America in the last few years because of the interest in extracting value from the stem of linseed varieties [4]. Stem fiber content does not seem associated with qualitative or quantitative plant characteristics in flax germplasm [4] indicating that there are no major biological restrictions for pyramiding agronomic and seed quality traits with high fiber content.

Crops have been subjected to strong selective pressure directed at genes controlling traits of agronomic importance during their domestication and subsequent episodes of selective breeding [47]. Under positive selection, favourable alleles will increase in frequency until fixation. As an effect of genetic hitchhiking, loci closely linked to beneficial alleles might present distortions from neutral expectations. Genome scans have allowed the identification of candidate loci involved in domestication and breeding traits in several crops [47,51] and domesticated animals [52,53]. However, population structure and bottlenecks can mimic the effect of selection and create false positives. The combination of several methods based on different assumptions can reduce false positives [54].

We applied four different tests of neutrality to identify the genomic regions that deviate from neutral expectations potentially associated with fiber and linseed divergent selection. Collectively, 86 candidate genes were identified at nine loci (Additional file 4: Table S2). Among our candidate genes, we found a β-tubulin involved in cell morphogenesis and elongation of fiber in cotton [55], a glucan endo-1,3-β-glucosidase associated with cell wall biogenesis/degradation in flax [56], a chitinase involved in polysaccharide degradation [56], a MYB transcription factor that influences cellulose microfibril angle in Eucalyptus [57] and a class III HD-Zip protein 4 (HB4) involved in xylem identity in flax [58] (Additional file 4: Table S2). Candidate genes such as pyruvate dehydrogenase E1 and fatty acid alpha-hydroxylase involved in fatty acid biosynthetic processes were also identified (Additional file 4: Table S2). However, β-galactosidase and cellulose synthase, two key enzymes for cell-wall modification and cellulose synthesis in flax [56,58] were not present at any of the nine loci. Previously identified genes in flax microarray analyses of hypocotyl and phloem fiber development [56] and differentially expressed genes between flax inner and outer stem tissues [58] were found among our candidate genes (Additional file 4: Table S2).

Although preliminary, our scans provided the first insights of non-neutral loci potentially affected by divergent selection in flax. Candidate genes, especially those previously reported [56,58], will require further investigation and validation. To enhance the probability of identifying additional candidate loci, a high density of markers would be desirable. Currently, next generation sequencing technology enables the re-sequencing of a large number of accessions at a reasonable price. Thus, high quality and dense single nucleotide polymorphism (SNP) markers promise to provide comprehensive genome coverage for the identification of non-neutral genomic regions in flax [53]. Such genomic tools for flax genetic studies are being developed and more comprehensive genomic scans will be possible in the near future.

## Conclusions

In this study, high levels of genetic diversity were revealed as compared to previous flax genetic studies. The weak population structure and relatedness and relatively fast LD decay indicate the suitability of this flax core collection for AM. The peculiar divergent breeding applied in the development of fiber and linseed flax varieties provides a unique opportunity to understand how human needs have sculpted the flax genome during domestication and improvement, and how these divergent genomic regions could be deployed in breeding for flax as a dual purpose crop.

## Methods

### Plant material

The PGRC flax collection has been evaluated in the field to measure seed characteristics, disease resistance and phenological traits [22]. Based on this information, a core collection of 381 flax accessions was assembled representing the phenotypic diversity of the PGRC flax world collection. To these, 26 accessions of relevance to recent Canadian flax breeding programs were added, resulting in a core collection of 407 accessions. Information on the geographic origin and improvement status of the accessions is shown (Additional file 6: Table S3 and Additional file 7: Figure S4). The core collection comprised 92 fiber accessions, 285 linseed accessions and 30 unknown accessions.

### DNA isolation and microsatellite genotyping

Genomic DNA was extracted from leaf tissues collected from a single plant of each accession [23]. DNA was quantified using a fluorometer and diluted to a 6 ng/μL working solution. Four hundred forty eight microsatellites [23,29,45,46,59] distributed across the 15 linkage groups [33] were analyzed following the procedure previously described [23]. Briefly, the amplification products were resolved on an ABI 3130xl Genetic analyzer (Applied Biosystems, Foster City, CA, USA). Output files were analyzed by GeneScan (Applied Biosystems) and subsequently imported into Genographer. Fragment sizes were estimated using GeneScan ROX-500 and MapMarker® 1000 (BioVentures Inc., Murfreesboro, TN) internal size standards, and the genotypic data matrix generated was used for all posterior analyses. The genotype of each locus was encoded based on its allele size in bp or as a null allele for dominant markers. The selective neutrality status was tested across microsatellites prior to other downstream genetic analyses using the Ewens-Watterson (EW) neutrality test [60] implemented in POPGENE v.1.31 [61] with 1,000 permutations without replacement.

### Phylogenetic analysis

To assess the genetic relationships among the accessions of the core collection, a dendrogram was generated using the neighbour-joining (NJ) algorithm [62] based on the Nei [62] minimum genetic distance method implemented in PowerMarker v.3.25 [63] and displayed by MEGA 5 [64]. The Nei [62] minimum genetic distance method is applicable to any population without regard to the number of alleles per locus, the pattern of evolutionary forces and the reproductive method of the organism studied. Thus it is a realistic estimation of the genetic relationships in an artificial population when individuals display different selection intensities, breeding objectives, and improvement status. The analysis was performed with the 414 neutral microsatellites identified by the EW neutrality test including minor allele frequency (MAF) < 0.05. The genotype of each marker was encoded as two alleles using their sizes estimated above as follow: homozygous state (allele1/allele1) and heterozygous state (allele1/allele2). Null alleles “null/null” were encoded as 999/999 and missing values as “?/?”. The reliability of the dendrogram topology was confirmed with 1,000 bootstraps with replacements.

### Population structure

To investigate the patterns of population structure, we conducted principal coordinate (PCoA) and Bayesian-based analyses. Because LD can affect both PCoA and STRUCTURE analyses, we thinned the marker set by excluding microsatellites in strong LD, i.e., markers with a square of the correlation coefficient (*r*^2^) greater than 0.4 [65]. Allelic frequencies were calculated in PowerMarker v.3.25 [63] and MAF < 0.05 were set to “U” (missing data) and excluded from the LD analysis. Genetic distances between markers were obtained from the microsatellite consensus linkage map of flax [33] integrated with the physical map [1]. Linked and unlinked LD (*r*^2^) was determined using GGT 2.0 [66] with genotypic data encoded as follows: 100/100 = A, 200/200 = B, 300/300 = C and so on, where each letter represents a different allele. Heterozygous individuals were considered missing value "U". PCoA was performed in a multidimensional space with data standardization using GENALEX v.6.41 [67]. Population structure analysis was carried out using STRUCTURE 2.3.3 [68,69]. The admixture model was used with a burn in of 10,000 and 100,000 iterations for *K* populations ranging from 1 to 12. Ten runs for each *K* value were performed and the ad-hoc statistic Δ*k* was used to determine the optimum number of sub-groups [70]. Prior to population structure analysis, SSR data was encoded using the size of each allele and “-9” was used for missing values. Accessions with estimated memberships ≥ 0.70 were assigned to corresponding groups; accessions with estimated memberships < 0.70 were assigned to a mixed group. We adopted a cut-off value of 0.70 because 85% of the accessions are cultivars and breeding material, thus it is likely that their genome structure resembles more than one ancestral population. The inferred sub-groups were visualized in Distruct [71]. Pairwise F_ST_ comparisons were calculated using GENALEX v.6.41 [67] to determine the genetic differentiation between the inferred genetic groups.

### Molecular coancestry

Strong familial relatedness can potentially inflate the number of spurious associations when it is not accounted for by the AM model. Relatedness was estimated using the molecular coancestry parameter (*f*_*ij*_) according to Caballero and Toro [72]. The molecular coancestry between two individuals *i* and *j* is the probability that two randomly sampled alleles from the same locus in two individuals are identical by state [72]. Molecular coancestry between two individuals *i* and *j* at a given locus can be computed using the following scoring rules [72]: *fij*,_*l*_ = ¼[*I*_11_ + *I*_12_ + *I*_21_ + *I*_22_], where *I*_*xy*_ is 1 when allele *x* on locus *l* in individual *i* and allele *y* in the same locus in individual *j* are identical and zero otherwise. Notice that this estimate can only have four values: 0, ¼, ½, and 1. The molecular coancestry between two individuals *i* and *j* (*fij*) can be obtained simply by averaging over *L* analyzed loci. Molecular coancestry matrices comparing all pairs of individuals within the core collection and within the different genetic groups identified above were calculated using all 448 microsatellites using MolKin v.3.0 [73]. Genotypic data based on the size of alleles was encoded as two alleles following the Genpop software format as follows: 100/200 = 0102, 200/200 = 0202 and so on. Missing values were labeled “0000”.

### Genetic diversity

Genetic diversity parameters were estimated across the genetic groups identified above based on the 414 neutral microsatellites. Unbiased gene diversity (UH_e_), observed heterozygosity (H_o_), total number of alleles (N_a_), inbreeding coefficient (F_IS_) and polymorphic loci (%) were calculated in GENALEX v.6.41 [67]. Allelic richness (R_s_) and private alleles (∏) were corrected for sample size differences and estimated using the rarefaction method implemented in HP-RARE v.1.2 [74]. The number of rare alleles (MAF < 0.05) and the polymorphism information content (PIC) values were calculated in PowerMarker v.3.25 [63].

### Linkage disequilibrium

LD was estimated by calculating *r*^2^ using GGT 2.0 [66] as described in the population structure section above. Only microsatellites with known chromosome information in the consensus map of flax [33] were used for LD estimation. Microsatellites on the same linkage group were considered linked and those on different linkage groups, unlinked. Mean LD was estimated for linked and unlinked markers in the total panel and for the different genetic groups identified by NJ and population structure analyses. The 95^th^ percentile of *r*^2^ distribution for unlinked markers was considered the cut-off LD value to determine whether LD resulted from physical linkage [75]. Average genome-wide LD decay versus genetic distance was estimated as previously described [75]. A cut-off value of *r*^2^ = 0.1 was set to estimate the average genome-wide LD block. In order to compare the trend of LD decay amongst the different genetic groups, we averaged LD values to distance intervals equal to the average genome-wide LD block estimated.

### Identification of non-neutral loci

To identify candidate loci linked to genomic regions that might have experienced divergent selection, we used the 92 fiber flax accessions present in the core collection (Additional file 6: Table S3) The “line selection” module in PowerMarker v.3.25 [63] allows the selection of a core set of lines from a large germplasm collection that maximizes the genetic diversity. Likewise, this module enables the selection of a random set of lines from a large population. Using PowerMarker v.3.25 [63] we randomly selected a set of 92 linseed accessions (among the 285 linseed accessions of the core collection) that captured the average number of alleles present in 100 random sets of 92 lines for the identification of non-neutral loci. Because bottlenecks can create false positive outliers, both fiber and linseed groups were analyzed with BOTTLENECK v.1.2.02 assuming the two-phase mutation model proposed for microsatellite data [38]. Genotypic data followed the Genepop format described above. We applied four outlier tests to minimize the number of false positives. (1) The Ewens-Watterson (EW) test statistic which identifies positively selected loci by evaluating significant deviation from expected heterozygosity (Dh/sd) in a single population [76] was calculated using BOTTLENECK v.1.2.02 [38]. Statistical significance (Dh/sd < −2.5, P < 0.05) was assigned based on 1,000 permutations without replacement. (2) The ln RH test that identifies loci that differ in variability from the remainder of the genome by calculating the ratio of gene diversity in two populations was performed [77]. After standardization of ln RH estimates, 95% of the neutral loci are expected to have values ranging between −1.96 and 1.96. Any locus with a value higher than 1.96 (P < 0.05) was considered non-neutral. (3) The Beaumont and Nichols [78] approach implemented in LOSITAN [79] identifies loci under selection based on the distribution of heterozygosity and F_ST_ under an island model of migration. The expected null distribution of F_ST_ values and estimated P values for each locus were obtained [79]. Loci exceeding the 95% upper confidence area were considered non-neutral. Genotypic data also followed the Genepop format described above. (4) The hierarchical island model that identifies outlier loci by allowing the exchange of more migrants within groups than between groups while generating the null distribution of F_ST_ values to reduce the number of false positives, was also applied to the data set [80]. The fiber and linseed groups were analyzed with STRUCTURE 2.3.3 [68,69] to determine the number of groups to incorporate in the hierarchical analysis using the ad-hoc statistic Δ*k*[70]. The expected F_ST_ distributions were obtained using Arlequin v.3.5 [81]. Loci outside the 95% upper confidence area were considered non-neutral (P < 0.05). The genotype of each marker was encoded as two alleles using their size estimate where the homozygous state was 100100 and the heterozygous state was 100200. Null alleles “null/null” were encoded as 999999 and missing values were “??”. Loci identified by at least two of the above four tests were retained and investigated as candidates for divergent selection.

### Candidate genes

To identify candidate genes by homology search, we used the combined information of the consensus genetic map [33], the physical map [1] and the whole genome shotgun (WGS) sequence assembly ([35]; http://www.phytozome.net) of flax. When the candidate locus and its adjacent marker with the highest LD (*r*^2^ > 0.4) were located in the same WGS sequence assembly scaffold, we estimated the physical to genetic distance (Mb/cM) to define the physical distance to be investigated for the identification of candidate genes. When adjacent markers were on different scaffolds or showed weak LD (*r*^2^ < 0.2), we limited the search for candidate genes to the 10 kb regions upstream and downstream of the outlier markers. Annotation of the WGS assembly using the Hidden Markov Model-based gene-finding programs Augustus v.2.5.5 [82] and GlimmerHMM v.3.0.1 [83] were used. Using the BLASTn algorithm, predicted open reading frames of candidate genes were searched against an in-house flax EST database comprising 462,190 flax ESTs ([23,34,58]; NCBI *Linum usitatissimum* ESTdb) for evidence of expression, using an E-value cutoff of 1e-5. The same candidate gene sequences were used to perform BLASTx searches against the 16 million annotated proteins in the UniProtKB db [84] to provide evidence of protein function using an E-value cutoff of 1e-5. Gene ontology (GO) annotations ([40]; http://www.geneontology.org) were also retrieved from the UniProtKB. Plant GO-slims for all three independent GO categories namely, cellular components, molecular functions and biological processes were obtained from all GO terms associated with the BLASTx gene annotations using the GO slim viewer from the AgBase web server ([85]; http://www.agbase.msstate.edu).

## Competing interests

The authors declare that they have no competing interests.

## Authors’ contributions

BJSC conducted this work as part of his PhD thesis. He carried out the analyses, interpretation of data and co-wrote the manuscript. AD characterized and developed the flax core collection. RR carried out the gene prediction and gene annotation. SC designed the study, generated the data, supervised the work and co-wrote the manuscript. All authors critically reviewed the manuscript. All authors read and approved the final manuscript.

## Supplementary Material

Additional file 1: Table S1 (Portable Document Format file) List of the 407 flax accessions sorted according to the neighbour-joining tree. Click here for file

Additional file 2: Figure S1 (Portable Document Format file) (**a**) Principal coordinate analysis (PCoA) of the 407 flax accessions of the core collection based on the 259 neutral SSRs with LD < 0.4. Sub-groups were labeled according to the NJ analysis results (Figure 1a). (**b**) Pairwise F_ST_ values between the 6 sub-groups of flax inferred by the NJ, STRUCTURE and PCoA analyses. 1 = North America. 2 = Eastern Europe. 3 = South Asia. 4 = Western Europe. 5 = North America/Europe. 6 = South America. * Significant values at P < 0.001. Click here for file

Additional file 3: Figure S2 Population structure and linkage disequilibrium analyses of the fiber flax and linseed groups (Portable Document Format file). (**a**) Bayesian clustering analysis (STRUCTURE *K* = 2) of fiber flax and linseed. (**b**) ad-hoc statistic Δ*K*[62] for *K* values ranging from 1 to 4. (**c**) Average genome-wide LD decay (*r*^2^) against genetic distance (cM) within fiber and linseed flax groups. The black line represents the decay curve at the genome level of the two flax groups. Click here for file

Additional file 4: Table S2 (Portable Document Format file) Analysis of candidate genes affected by divergent selection between fiber flax and linseed groups. green: BLASTx hit vs UniProtKB (No Hits), blue: BLASTx hit vs UniProtKB (less than 34 aminoacids or 35% similarity), red: BLASTn hit against Flax-ESTs (No Hits), yellow: BLASTn hit against Flax-ESTs (less than 80 bp or 80% similarity). Click here for file

Additional file 5: Figure S3 (Portable Document Format file) GO-slim annotations of gene products predicted from nine non-neutral candidate genomic regions between fiber flax and linseed groups. (**a**) Molecular function. (**b**) Biological process. (**c**) Cellular component. Click here for file

Additional file 6: Table S3 (Portable Document Format file) Core collection data including accession number, accession name, origin and improvement status. CN = Canadian number, Plant Gene Resources of Canada (PGRC). Click here for file

Additional file 7: Figure S4 (Portable Document Format file) Distribution of the 407 flax accessions of the core collection. (**a**) geographical origin. (**b**) improvement status. Click here for file
