# Supplementary material for: Genetic characterization of a core collection of flax (Linum usitatissimum L.) suitable for association mapping studies and evidence of divergent selection between fiber and linseed types
Source: BMC Plant Biol. 2013 May 6;13:78. doi: 10.1186/1471-2229-13-78 (PMC3656786; doi:10.1186/1471-2229-13-78)
Supplement: Additional file 1: Table S1 — (Portable Document Format file) List of the 407 flax accessions sorted according to the neighbour-joining tree. [file 1471-2229-13-78-S1.pdf]

**Table S1** List of the 407 flax accessions sorted according to the neighbour-joining tree

| Number | Canadian number | Sub-group |
|---|---|---|
| 120 | **CN97605** | Western Europe |
| 261 | **CN100837** | Western Europe |
| 274 | **CN100910** | Western Europe |
| 266 | **CN100852** | Western Europe |
| 272 | **CN100885** | Western Europe |
| 65 | **CN97147** | Western Europe |
| 368 | **CN101554** | Western Europe |
| 361 | **CN101496** | Western Europe |
| 269 | **CN100881** | Western Europe |
| 270 | **CN100883** | Western Europe |
| 199 | **CN98535** | Western Europe |
| 70 | **CN97238** | Western Europe |
| 219 | **CN98741** | Western Europe |
| 184 | **CN98278** | Western Europe |
| 46 | **CN96911** | Western Europe |
| 217 | **CN98733** | Western Europe |
| 218 | **CN98734** | Western Europe |
| 371 | **CN101565** | Western Europe |
| 216 | **CN98712** | Western Europe |
| 222 | **CN98753** | Western Europe |
| 375 | **CN101595** | Western Europe |
| 170 | **CN98192** | Western Europe |
| 171 | **CN98193** | Western Europe |
| 223 | **CN98767** | Western Europe |
| 114 | **CN97571** | Western Europe |
| 262 | **CN100838** | Western Europe |
| 77 | **CN97321** | Western Europe |
| 185 | **CN98279** | Western Europe |
| 304 | **CN101265** | Western Europe |
| 248 | **CN100674** | Western Europe |
| 71 | **CN97287** | Western Europe |
| 275 | **CN100928** | Western Europe |
| 280 | **CN101026** | Western Europe |
| 197 | **CN98475** | Western Europe |
| 235 | **CN98923** | Western Europe |
| 187 | **CN98303** | Western Europe |
| 388 | **CrepitamTabor** | Western Europe |
| 320 | **CN101338** | South Asia |
| 66 | **CN97153** | South Asia |
| 325 | **CN101373** | South Asia |
| 168 | **CN98165** | South Asia |
| 48 | **CN96962** | South Asia |
| 256 | **CN100799** | South Asia |
| 62 | **CN97129** | South Asia |

| | | |
|---|---|---|
| 260 | **CN100828** | South Asia |
| 63 | **CN97129B** | South Asia |
| 378 | **CN101600** | South Asia |
| 257 | **CN100805** | South Asia |
| 263 | **CN100841** | South Asia |
| 252 | **CN100790** | South Asia |
| 45 | **CN96846** | South Asia |
| 259 | **CN100827** | South Asia |
| 314 | **CN101310** | South Asia |
| 169 | **CN98176** | South Asia |
| 198 | **CN98505** | South Asia |
| 233 | **CN98869** | South Asia |
| 20 | **CN19007** | South Asia |
| 53 | **CN97004** | South Asia |
| 51 | **CN96991** | South Asia |
| 52 | **CN96992** | South Asia |
| 358 | **CN101482** | South Asia |
| 50 | **CN96988** | South Asia |
| 356 | **CN101471** | South Asia |
| 357 | **CN101472** | South Asia |
| 44 | **CN96845** | South Asia |
| 85 | **CN97393** | South Asia |
| 299 | **CN101208** | South Asia |
| 76 | **CN97312** | South Asia |
| 74 | **CN97307** | South Asia |
| 75 | **CN97308** | South Asia |
| 204 | **CN98566C** | South Asia |
| 202 | **CN98566** | South Asia |
| 203 | **CN98566B** | South Asia |
| 73 | **CN97306** | South Asia |
| 167 | **CN98157** | South Asia |
| 205 | **CN98569** | South Asia |
| 61 | **CN97103** | South Asia |
| 59 | **CN97092** | South Asia |
| 60 | **CN97096** | South Asia |
| 200 | **CN98541** | South Asia |
| 207 | **CN98613** | South Asia |
| 206 | **CN98610** | South Asia |
| 376 | **CN101596** | South Asia |
| 244 | **CN98982** | South Asia |
| 364 | **CN101535** | South Asia |
| 365 | **CN101536** | South Asia |
| 366 | **CN101539** | South Asia |
| 195 | **CN98467** | South Asia |
| 196 | **CN98468** | South Asia |
| 194 | **CN98440** | South Asia |
| 47 | **CN96958** | South Asia |

| | | |
|---|---|---|
| 174 | **CN98239** | South Asia |
| 247 | **CN100629** | South Asia |
| 58 | **CN97083** | South Asia |
| 54 | **CN97050** | South Asia |
| 362 | **CN101510** | South Asia |
| 363 | **CN101511** | South Asia |
| 178 | **CN98250** | South Asia |
| 56 | **CN97064** | South Asia |
| 55 | **CN97056** | South Asia |
| 188 | **CN98363** | South Asia |
| 180 | **CN98263** | South Asia |
| 181 | **CN98263B** | South Asia |
| 57 | **CN97072** | South Asia |
| 67 | **CN97176** | South Asia |
| 49 | **CN96974** | South Asia |
| 360 | **CN101493** | South Asia |
| 173 | **CN98237** | South Asia |
| 232 | **CN98854** | South Asia |
| 249 | **CN100678** | South Asia |
| 354 | **CN101466** | South Asia |
| 165 | **CN98135** | South Asia |
| 191 | **CN98397** | South Asia |
| 189 | **CN98364** | South Asia |
| 64 | **CN97139** | South Asia |
| 193 | **CN98415** | South Asia |
| 161 | **CN98057** | South Asia |
| 241 | **CN98969** | South Asia |
| 164 | **CN98109** | South Asia |
| 367 | **CN101542** | South Asia |
| 192 | **CN98398** | South Asia |
| 242 | **CN98973** | South Asia |
| 179 | **CN98254** | South Asia |
| 243 | **CN98974** | South Asia |
| 190 | **CN98370** | South Asia |
| 177 | **CN98242** | South Asia |
| 240 | **CN98961** | South Asia |
| 175 | **CN98240** | South Asia |
| 176 | **CN98240B** | South Asia |
| 377 | **CN101598** | South America |
| 12 | **CN18993** | South America |
| 403 | **UGG102-2** | South America |
| 389 | **DoubleLow** | South America |
| 404 | **UGG146-1** | South America |
| 151 | **CN97980** | South America |
| 152 | **CN98007** | South America |
| 118 | **CN97587** | South America |
| 156 | **CN98037** | South America |

| | | |
|---|---|---|
| 157 | **CN98037B** | South America |
| 150 | **CN97967** | South America |
| 158 | **CN98039** | South America |
| 149 | **CN97961** | South America |
| 147 | **CN97953** | South America |
| 148 | **CN97958** | South America |
| 212 | **CN98689** | South America |
| 124 | **CN97633** | South America |
| 208 | **CN98634** | South America |
| 163 | **CN98100** | South America |
| 95 | **CN97430** | South America |
| 96 | **CN97430B** | South America |
| 353 | **CN101463** | South America |
| 153 | **CN98012** | South America |
| 155 | **CN98027** | South America |
| 209 | **CN98639** | North America-Europe |
| 210 | **CN98644** | North America-Europe |
| 182 | **CN98275** | North America-Europe |
| 183 | **CN98276** | North America-Europe |
| 245 | **CN98984** | North America-Europe |
| 224 | **CN98773** | North America-Europe |
| 117 | **CN97586** | North America-Europe |
| 221 | **CN98752** | North America-Europe |
| 374 | **CN101594** | North America-Europe |
| 373 | **CN101580** | North America-Europe |
| 82 | **CN97366** | North America-Europe |
| 254 | **CN100797** | North America-Europe |
| 255 | **CN100797B** | North America-Europe |
| 213 | **CN98704** | North America-Europe |
| 25 | **CN19160** | North America-Europe |
| 69 | **CN97214** | North America-Europe |
| 130 | **CN97670** | North America-Europe |
| 144 | **CN97890** | North America-Europe |
| 145 | **CN97907** | North America-Europe |
| 201 | **CN98542** | North America-Europe |
| 172 | **CN98231** | North America-Europe |
| 359 | **CN101486** | North America-Europe |
| 258 | **CN100807** | North America-Europe |
| 279 | **CN101016** | North America-Europe |
| 319 | **CN101332** | North America-Europe |
| 379 | **CN101610** | North America-Europe |
| 295 | **CN101132** | North America-Europe |
| 305 | **CN101279** | North America-Europe |
| 338 | **CN101402** | North America-Europe |
| 26 | **CN30860** | North America-Europe |
| 27 | **CN30861** | North America-Europe |
| 226 | **CN98806** | North America-Europe |

| | | |
|---|---|---|
| 227 | **CN98807** | North America-Europe |
| 214 | **CN98708** | North America-Europe |
| 372 | **CN101572** | North America-Europe |
| 268 | **CN100864** | North America-Europe |
| 68 | **CN97180** | North America-Europe |
| 24 | **CN19159** | North America-Europe |
| 355 | **CN101469** | North America-Europe |
| 72 | **CN97300** | North America-Europe |
| 80 | **CN97350** | North America-Europe |
| 154 | **CN98014** | North America-Europe |
| 220 | **CN98742** | North America-Europe |
| 134 | **CN97689** | North America |
| 140 | **CN97871** | North America |
| 265 | **CN100851** | North America |
| 271 | **CN100884** | North America |
| 297 | **CN101137** | North America |
| 312 | **CN101307** | North America |
| 352 | **CN101461** | North America |
| 370 | **CN101560** | North America |
| 228 | **CN98812** | North America |
| 277 | **CN100939** | North America |
| 324 | **CN101367** | North America |
| 139 | **CN97768** | North America |
| 133 | **CN97679B** | North America |
| 132 | **CN97679** | North America |
| 142 | **CN97881** | North America |
| 79 | **CN97341** | North America |
| 93 | **CN97407** | North America |
| 100 | **CN97458** | North America |
| 225 | **CN98794** | North America |
| 131 | **CN97671** | North America |
| 38 | **CN33400** | North America |
| 17 | **CN19003** | North America |
| 141 | **CN97873** | North America |
| 32 | **CN33388** | North America |
| 10 | **CN18989** | North America |
| 33 | **CN33389** | North America |
| 251 | **CN100785** | North America |
| 39 | **CN33992** | North America |
| 1 | **CN18973** | North America |
| 399 | **PrairieGrande** | North America |
| 400 | **PrairieThunder** | North America |
| 383 | **Shape** | North America |
| 382 | **Macbeth** | North America |
| 391 | **FP2214** | North America |
| 18 | **CN19004** | North America |
| 384 | **CDCSorrel** | North America |

| | | |
|---|---|---|
| 344 | **CN101413** | North America |
| 4 | **CN18981** | North America |
| 19 | **CN19005** | North America |
| 30 | **CN33385** | North America |
| 3 | **CN18980** | North America |
| 43 | **CN52732** | North America |
| 21 | **CN19017** | North America |
| 28 | **CN32542** | North America |
| 250 | **CN100770** | North America |
| 78 | **CN97334** | North America |
| 135 | **CN97718** | North America |
| 137 | **CN97740** | North America |
| 329 | **CN101382** | North America |
| 136 | **CN97728** | North America |
| 138 | **CN97749** | North America |
| 146 | **CN97921** | North America |
| 229 | **CN98821** | North America |
| 127 | **CN97642** | North America |
| 37 | **CN33399** | North America |
| 395 | **Lirina** | North America |
| 331 | **CN101386** | North America |
| 13 | **CN18994** | North America |
| 36 | **CN33397** | North America |
| 392 | **FP2270** | North America |
| 386 | **CDCBethune** | North America |
| 387 | **CDCMons** | North America |
| 2 | **CN18979** | North America |
| 398 | **PrairieBlue** | North America |
| 396 | **M5791** | North America |
| 405 | **UGG5-5** | North America |
| 393 | **G1186-94** | North America |
| 402 | **S95407** | North America |
| 41 | **CN37286** | North America |
| 381 | **CDCGold** | North America |
| 407 | **YSED18** | North America |
| 380 | **Linola989** | North America |
| 401 | **SP2047** | North America |
| 394 | **Hanley** | North America |
| 390 | **E1747** | North America |
| 397 | **M96006** | North America |
| 323 | **CN101366** | Eastern Europe |
| 330 | **CN101385** | Eastern Europe |
| 98 | **CN97452** | Eastern Europe |
| 273 | **CN100895B** | Eastern Europe |
| 326 | **CN101375** | Eastern Europe |
| 84 | **CN97392** | Eastern Europe |
| 315 | **CN101325** | Eastern Europe |

| | | |
|---|---|---|
| 318 | **CN101331** | Eastern Europe |
| 186 | **CN98286** | Eastern Europe |
| 122 | **CN97613** | Eastern Europe |
| 278 | **CN100952** | Eastern Europe |
| 246 | **CN100547** | Eastern Europe |
| 31 | **CN33386** | Eastern Europe |
| 385 | **Atlas** | Eastern Europe |
| 81 | **CN97351** | Eastern Europe |
| 336 | **CN101397** | Eastern Europe |
| 119 | **CN97604** | Eastern Europe |
| 92 | **CN97406** | Eastern Europe |
| 328 | **CN101379** | Eastern Europe |
| 316 | **CN101327** | Eastern Europe |
| 321 | **CN101348** | Eastern Europe |
| 104 | **CN97483** | Eastern Europe |
| 128 | **CN97649** | Eastern Europe |
| 327 | **CN101378** | Eastern Europe |
| 83 | **CN97377** | Eastern Europe |
| 313 | **CN101308** | Eastern Europe |
| 87 | **CN97397** | Eastern Europe |
| 89 | **CN97403** | Eastern Europe |
| 99 | **CN97453** | Eastern Europe |
| 125 | **CN97639** | Eastern Europe |
| 126 | **CN97639B** | Eastern Europe |
| 86 | **CN97396** | Eastern Europe |
| 88 | **CN97402** | Eastern Europe |
| 90 | **CN97404** | Eastern Europe |
| 91 | **CN97404B** | Eastern Europe |
| 215 | **CN98710** | Eastern Europe |
| 237 | **CN98934** | North America |
| 306 | **CN101286** | North America |
| 350 | **CN101451** | North America |
| 22 | **CN19157** | North America |
| 267 | **CN100863** | North America |
| 349 | **CN101448** | North America |
| 94 | **CN97424** | North America |
| 369 | **CN101559** | North America |
| 236 | **CN98926** | North America |
| 239 | **CN98954** | North America |
| 211 | **CN98683** | North America |
| 238 | **CN98946** | North America |
| 162 | **CN98072** | North America |
| 23 | **CN19158** | North America |
| 264 | **CN100848** | North America |
| 351 | **CN101454** | North America |
| 115 | **CN97584** | North America |
| 116 | **CN97584B** | North America |

| | | |
|---|---|---|
| 231 | **CN98829** | North America |
| 302 | **CN101240** | Eastern Europe |
| 105 | **CN97484** | Eastern Europe |
| 343 | **CN101407** | Eastern Europe |
| 110 | **CN97529** | Eastern Europe |
| 121 | **CN97610** | Eastern Europe |
| 111 | **CN97530** | Eastern Europe |
| 253 | **CN100795** | Eastern Europe |
| 109 | **CN97520** | Eastern Europe |
| 123 | **CN97616** | Eastern Europe |
| 97 | **CN97444** | Eastern Europe |
| 101 | **CN97463** | Eastern Europe |
| 230 | **CN98826** | Eastern Europe |
| 106 | **CN97487** | Eastern Europe |
| 107 | **CN97489** | Eastern Europe |
| 113 | **CN97533** | Eastern Europe |
| 108 | **CN97503** | Eastern Europe |
| 129 | **CN97665** | Eastern Europe |
| 143 | **CN97886** | Eastern Europe |
| 322 | **CN101364** | Eastern Europe |
| 112 | **CN97531** | Eastern Europe |
| 102 | **CN97470** | Eastern Europe |
| 103 | **CN97475** | Eastern Europe |
| 234 | **CN98903** | Eastern Europe |
| 159 | **CN98056** | Eastern Europe |
| 160 | **CN98056B** | Eastern Europe |
| 166 | **CN98150** | Eastern Europe |
| 301 | **CN101237** | Eastern Europe |
| 332 | **CN101392** | Eastern Europe |
| 283 | **CN101052** | Eastern Europe |
| 284 | **CN101053** | Eastern Europe |
| 7 | **CN18986** | Eastern Europe |
| 406 | **Viking(European)** | Eastern Europe |
| 8 | **CN18987** | Eastern Europe |
| 5 | **CN18982** | Eastern Europe |
| 9 | **CN18988** | Eastern Europe |
| 341 | **CN101405** | Eastern Europe |
| 298 | **CN101154** | Eastern Europe |
| 293 | **CN101119** | Eastern Europe |
| 35 | **CN33393** | Eastern Europe |
| 276 | **CN100929** | Eastern Europe |
| 6 | **CN18983** | Eastern Europe |
| 11 | **CN18991** | Eastern Europe |
| 16 | **CN19001** | Eastern Europe |
| 15 | **CN18998** | Eastern Europe |
| 34 | **CN33390** | Eastern Europe |
| 42 | **CN40081** | Eastern Europe |

| | | |
|---|---|---|
| 339 | **CN101403** | Eastern Europe |
| 317 | **CN101329** | Eastern Europe |
| 340 | **CN101404** | Eastern Europe |
| 29 | **CN32546** | Eastern Europe |
| 296 | **CN101136** | Eastern Europe |
| 14 | **CN18997** | Eastern Europe |
| 307 | **CN101289** | Eastern Europe |
| 285 | **CN101055** | Eastern Europe |
| 40 | **CN35791** | Eastern Europe |
| 310 | **CN101299** | Eastern Europe |
| 342 | **CN101406** | Eastern Europe |
| 348 | **CN101421** | Eastern Europe |
| 345 | **CN101416** | Eastern Europe |
| 347 | **CN101419** | Eastern Europe |
| 300 | **CN101230** | Eastern Europe |
| 346 | **CN101417** | Eastern Europe |
| 281 | **CN101038** | Eastern Europe |
| 286 | **CN101094** | Eastern Europe |
| 291 | **CN101116** | Eastern Europe |
| 337 | **CN101401** | Eastern Europe |
| 292 | **CN101118** | Eastern Europe |
| 333 | **CN101394** | Eastern Europe |
| 287 | **CN101096** | Eastern Europe |
| 288 | **CN101099** | Eastern Europe |
| 309 | **CN101298** | Eastern Europe |
| 311 | **CN101301** | Eastern Europe |
| 282 | **CN101039** | Eastern Europe |
| 294 | **CN101127** | Eastern Europe |
| 335 | **CN101396** | Eastern Europe |
| 334 | **CN101395** | Eastern Europe |
| 308 | **CN101296** | Eastern Europe |
| 290 | **CN101115** | Eastern Europe |
| 289 | **CN101114** | Eastern Europe |
| 303 | **CN101241** | Eastern Europe |
