# Supplementary material for: Genetic characterization of a core collection of flax (Linum usitatissimum L.) suitable for association mapping studies and evidence of divergent selection between fiber and linseed types
Source: BMC Plant Biol. 2013 May 6;13:78. doi: 10.1186/1471-2229-13-78 (PMC3656786; doi:10.1186/1471-2229-13-78)
Supplement: Additional file 2: Figure S1 — (Portable Document Format file) (a) Principal coordinate analysis (PCoA) of the 407 flax accessions of the core collection based on the 259 neutral SSRs with LD < 0.4. Sub-groups were labeled according to the NJ analysis results (Figure 1a). (b) Pairwise FST values between the 6 sub-groups of flax inferred by the NJ, STRUCTURE and PCoA analyses. 1 = North America. 2 = Eastern Europe. 3 = South Asia. 4 = Western Europe. 5 = North America/Europe. 6 = South America. * Significant values at P < 0.001. [file 1471-2229-13-78-S2.pdf]

(a)

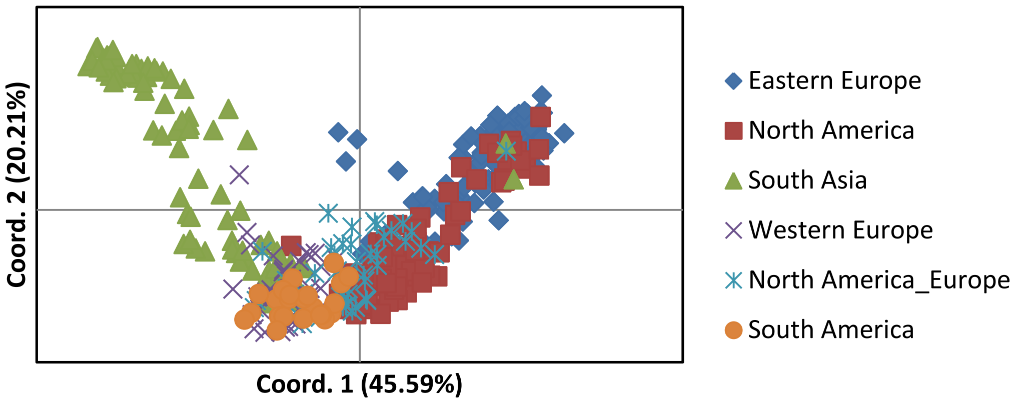

(b)

	1	2	3	4	5	6
1	—					
2	0.02*	—				
3	0.13*	0.16*	—			
4	0.08*	0.12*	0.07*	—		
5	0.04*	0.07*	0.09*	0.04*	—	
6	0.08*	0.12*	0.11*	0.05*	0.04*	—

Figure S1. (a) Principal coordinate analysis (PCoA) of the 407 flax accessions of the core collection based on the 259 neutral SSRs with LD < 0.4. Sub-groups were labeled according to the NJ analysis results (Figure 1a). **(b)** Pairwise F_{ST} values between the 6 sub-groups of flax inferred by the NJ, STRUCTURE and PCoA analyses. 1 = North America. 2 = Eastern Europe. 3 = South Asia. 4 = Western Europe. 5 = North America/Europe. 6 = South America. * Significant values at P < 0.001.
