# Supplementary material for: Genetic characterization of a core collection of flax (Linum usitatissimum L.) suitable for association mapping studies and evidence of divergent selection between fiber and linseed types
Source: BMC Plant Biol. 2013 May 6;13:78. doi: 10.1186/1471-2229-13-78 (PMC3656786; doi:10.1186/1471-2229-13-78)
Supplement: Additional file 3: Figure S2 — Population structure and linkage disequilibrium analyses of the fiber flax and linseed groups (Portable Document Format file). (a) Bayesian clustering analysis (STRUCTURE K = 2) of fiber flax and linseed. (b) ad-hoc statistic ΔK[62] for K values ranging from 1 to 4. (c) Average genome-wide LD decay (r2) against genetic distance (cM) within fiber and linseed flax groups. The black line represents the decay curve at the genome level of the two flax groups. [file 1471-2229-13-78-S3.pdf]

(a)

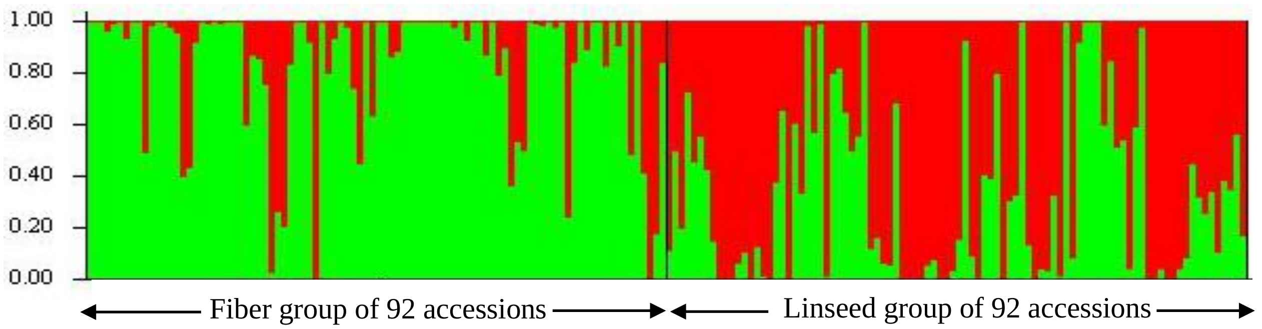

(b)

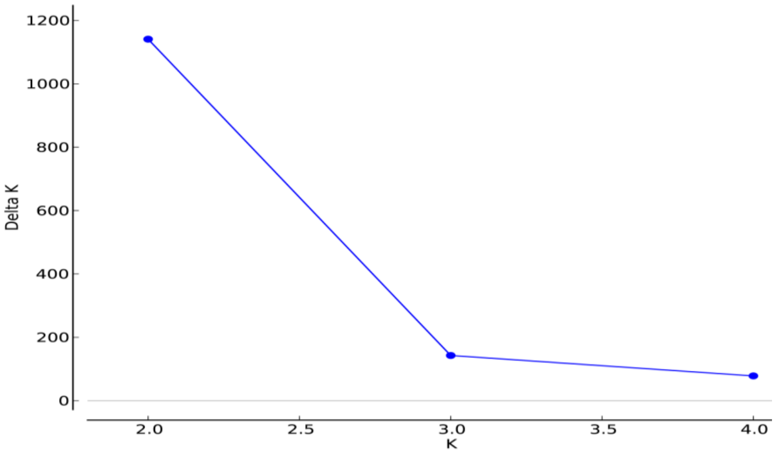

(c)

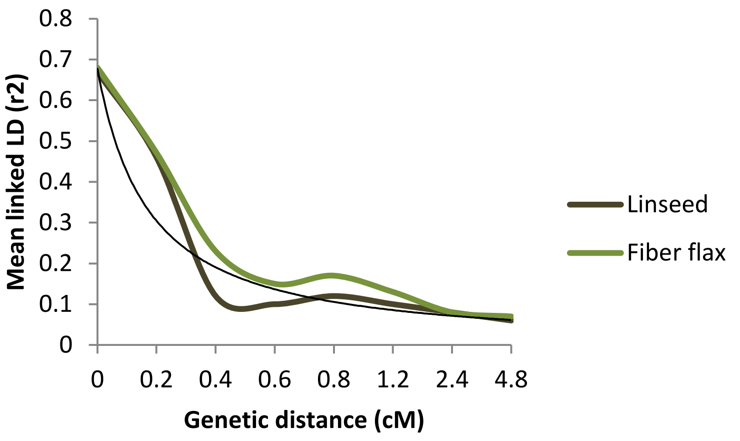

Figure S2. Population structure and linkage disequilibrium analyses of the fiber flax and linseed groups (Portable Document Format file). **(a)** Bayesian clustering analysis (STRUCTURE $K = 2$) of fiber flax and linseed. **(b)** *ad-hoc* statistic ΔK [62] for K values ranging from 1 to 4. **(c)** Average genome-wide LD decay (r^2) against genetic distance (cM) within fiber and linseed flax groups. The black line represents the decay curve at the genome level of the two flax groups.
