# Supplementary material for: Genetic characterization of a core collection of flax (Linum usitatissimum L.) suitable for association mapping studies and evidence of divergent selection between fiber and linseed types
Source: BMC Plant Biol. 2013 May 6;13:78. doi: 10.1186/1471-2229-13-78 (PMC3656786; doi:10.1186/1471-2229-13-78)
Supplement: Additional file 4: Table S2 — (Portable Document Format file) Analysis of candidate genes affected by divergent selection between fiber flax and linseed groups. green: BLASTx hit vs UniProtKB (No Hits), blue: BLASTx hit vs UniProtKB (less than 34 aminoacids or 35% similarity), red: BLASTn hit against Flax-ESTs (No Hits), yellow: BLASTn hit against Flax-ESTs (less than 80 bp or 80% similarity). [file 1471-2229-13-78-S4.pdf]

Table S2 Analysis of candidate genes affected by divergent selection between fiber flax and linseed groups

Si.No	Query	BLASTx-Hit (Against UniProtKB database)	alignment length	Identity %	UniPROTKB ID	GI number	GO Id	*Blastn hit against Flax-ESTs*	*Reference*
1	s156-gene1-Lus10040693class=Sequence position=scaffold156:1042107..1044578 (+ strand	Full=50S ribosomal protein L4, chloroplastic;	165/219 (75%)	4e-106	O50061.2	GI:21542429	GO:0009570, GO:0009535, GO:0022626,GO:0005634, GO:0000311, GO:0008266, GO:0019843, GO:0003735,	*gb|JG259492 LUSTE1AD-RP-275_N17_20MAY2008_067 LUSTE1AD Linum usitatissimum cDNA,mRNA sequence Length = 869;Expect = 0.0 Identities = 823/826 (99%)*	
2	s156-gene-2-Lus10040694-class=Sequence position=scaffold156:1044953..1047480 (- strand)	Full=Phosphomethylpyrimidine synthase, chloroplastic;	261/278 (94%)	0	O82392.1	GI:75220243	GO:0051536, GO:0016829, GO:0046872, GO:0010266, GO:0009228, GO:0009229,	*gb|JG218635.1|JG218635 LUSST1AD-UP-101_E02_16JULY2008_008 LUSST1AD Linum usitatissimum cDNA, mRNA sequence Length = 779,Expect = 0.0 , Identities = 719/721 (99%), Gaps = 1/721 (0%)*	
3	s156-Gene3-Lus10040695 class=Sequence position=scaffold156:1048891..1053637 (- strand)	Full=ATP-dependent zinc metalloprotease FTSH 7, chloroplastic;	139/239 (58%)	1e-63	Q6H6R9.1	GI:75323554	GO:0016021, GO:0005524, GO:0004222, GO:0017111, GO:0008270,GO:0030163, GO:0006508,	*genolin_c27713 338 nt similar to |AY149938|AY149938|Arabidopsis thaliana;At2g47010/F14M4.16 mRNA, complete cds;Length = 1960; Expect = e-118; Identities = 218/219 (99%)*	
4	s156-Gene4-Lus10040696 class=Sequence position=scaffold156:1064047..1065045 (+ strand)	Full=Transcription factor MYB1R1;	58/120 (48%)	8e-25	Q2V9B0.1	GI:122232932	GO:0005829, GO:0005634, GO:0003677, GO:0006355, GO:0006950, GO:0006351,	*genolin_c20781 195 nt Length = 1716; Expect = 0.0; Identities = 481/485 (99%)*	Cellulose microfibril angle, wood collapse in Eucaliptus piluraris (Sexton et al. 2011)
5	s156-Gene5-Lus10040697 class=Sequence position=scaffold156:1068694..1071500 (- strand)	Full=Benzenediol:oxygen oxidoreductase 22;	231/390 (59%)	0	Q0IQU1.2	GI:150383842	GO:0048046, GO:0005507, GO:0052716, GO:0046274, GO:0009834,	*gb|JG063140.1|JG063140 LUSES3AD-T3-001_D12_10AUG2009_046 LUSES1AD Linum usitatissimum cDNA, mRNA sequence Length = 855, Expect = 0.0, Identities = 487/488 (99%)*	
6	s291-Gene1-Lus10032405 class=Sequence position=scaffold291:1153041..1155600 (+ strand)	RecName: Full=Uncharacterized protein SLP1; AltName: Full=SUN-like protein 1; Flags: Precursor Length=587	105/345 (30%)	2e-33	Q12232.1	GI:74676556	GO:0016021, GO:0034975,	*genolin_c34845 286 nt Length = 286 Expect = e-133 Identities = 277/286 (96%), Gaps = 2/286 (0%)*	
7	s291-Gene2-Lus10032406 class=Sequence position=scaffold291:1156965..1157978 (+ strand)	Full=Cysteine endopeptidase; Flags: Precursor	195/342 (57%)	1e-125	O65039.1	GI:46395620	GO:0016023,GO:0008234, GO:0006508,GO:0005737, GO:0005524, GO:0004829, GO:0006435,	*gb|JG214310.1|JG214310 LUSPS1AD_RP_105_I10_14AUG2008_039 LUSPS1AD Linum usitatissimum cDNA, mRNA sequence Length = 856 Score = 58.0 bits (29), Expect = 9e-007 Identities = 62/73 (84%)*	expressed stem inner tissue (Fenart et al. 2010)
8	s291-Gene3-Lus10032407 class=Sequence position=scaffold291:1159246..1161226 (+ strand)	Full=F-box protein SKIP8; AltName: Full=SKP1-interacting partner 8	53/71 (75%)	8e-30	Q93YV9.1	GI:75249436	GO:0016567,	*genolin_c35105 388 nt highly similar to |AL161518 Arabidopsis thaliana DNA chromosome 4, contig fragment No. 30 |Length = 738, Expect = e-163 Identities = 356/376 (94%), Gaps = 1/376 (0%)*	

9	s291-Gene4-Lus10032408 class=Sequence position=scaffold291:1162354..1163193 (+ strand)		No Hits found					*gb|JG032954.1|JG032954 03-LUSBE1NG-RP-203_H12_31MAR2007_082 LUSBE1NG Linum usitatissimum cDNA, mRNA sequence Length = 653, Expect = e-157 Identities = 281/281 (100%)*
10	s291-Gene5-Lus10032409 class=Sequence position=scaffold291:1164172..1164444 (+ strand)	Full=RNA polymerase II transcriptional coactivator KELP Length=165	26/55 (47%)	3e-8	O65155.1	GI:37079408	GO:0005634,GO:0003677, GO:0003713, GO:0006355, GO:0006351,	*USHE1NG_RP_041_E05_09FEB2007_039.ab1; Length = 530; Expect = e-153; Identities = 273/273 (100%)*
11	s291-Gene6-Lus10032410 class=Sequence position=scaffold291:1165833..1167092 (+ strand)	Full=RNA polymerase II transcriptional coactivator KELP Length=165	75/171 (44%),	3e-26	O65155.1	GI:37079408	GO:0005634, GO:0003677, GO:0003713, GO:0006355, GO:0006351,	*gb|JG263915.1|JG263915 LUSTE1AD-RP-289_F05_22MAY2008_027 LUSTE1AD Linum usitatissimum cDNA, mRNA sequence Length = 817, Expect = e-168, Identities = 302/303 (99%)*
12	s291-Gene7-Lus10032411 class=Sequence position=scaffold291:1167483..1168082 (- strand)							*genolin_c28591 232 nt Length = 797, Expect = 6e-096 Identities = 178/178 (100%)*
13	s291-Gene8-Lus10032412 class=Sequence position=scaffold291:1171667..1173288 (- strand)	Full=B2 protein Length=207	60/69 (87%	2e-34	P37707.1	GI:584825	No ontology	*gb|JG226758.1|JG226758 LUSST4AD-T3-041_M18_15SEP2009_067 LUSST1AD Linum usitatissimum cDNA, mRNA sequence Length = 792, Expect = 0.0,Identities = 430/432 (99%)*
14	s917-Gene1-Lus10030641 class=Sequence position=scaffold917:1149514..1149813 (- strand)	No Hit						*genolin_c33219 350 nt,Length = 1666,Expect = 2e-048, Identities = 122/130 (93%)*
15	s917-Gene2-Lus10030642 class=Sequence position=scaffold917:1159087..1159546 (+ strand)	Full=Probable threonine--tRNA ligase, cytoplasmic; AltName: Full=Threonyl-tRNA synthetase; Short=ThrRS	28/52 (54%)	1e-7	Q8GZ45.2	GI:85701287	GO:0005737, GO:0005524, GO:0004829,GO:0006435,	*gb|CA482850.1|CA482850 LuP12001G08R LuP12 Linum usitatissimum cDNA clone LuP12001G08R,mRNA sequence, Length = 637,Expect = 2e-015, Identities = 106/127 (83%)*
16	s917-Gene3-Lus10030643 class=Sequence position=scaffold917:1160394..1162880 (- strand)	Full=Kinetochore protein NDC80 homolog; AltName: Full=Kinetochore protein Hec1; AltName: Full=Kinetochore-associated protein 2 Length=642	67/267 (25%)	8e-17	Q9D0F1.1	GI:81881154	GO:0000777, GO:0000942, GO:0031262, GO:0008608, GO:0051301, GO:0007059, GO:0000132, GO:0007067, GO:0007052,	*genolin_c14408 482 nt,Length = 1805, Expect = 0.0,Identities = 470/480 (97%), Gaps = 4/480 (0%)*
17	s917-Gene4-Lus10030644 class=Sequence position=scaffold917:1163157..1163716 (+ strand)	Full=Threonine--tRNA ligase, mitochondrial; AltName: Full=Threonyl-tRNA synthetase; Short=ThrRS; Flags: Precursor	42/75 (56%),	8e-26	O04630.3	GI:27735258	GO:0005618,GO:0009507, GO:0005829, GO:0005739, GO:0005886, GO:0005524,GO:0004829, GO:0006435,	*gb|JG151717.1|JG151717 LUSHE1NG-RP-076_F05_15FEB2007_037 LUSHE1NG Linum usitatissimum cDNA, mRNA sequence Length = 641,Expect = 3e-017, Identities = 103/122 (84%)*

18	s917-Gene5-Lus10030645 class=Sequence position=scaffold917:1163873..1170020 (+ strand)	RecName: Full=Threonine--tRNA ligase, mitochondrial; AltName: Full=Threonyl-tRNA synthetase; Short=ThrRS; Flags: Precursor Length=709	79/132 (60%)	9e-86	O04630.3	GI:27735258	GO:0005618, GO:0009507, GO:0005829, GO:0005739, GO:0005886, GO:0005524, GO:0004829, GO:0006435,	*genolin_c27668 527 nt Length = 1400, Expect = 1e-059 Identities = 167/182 (91%), Gaps = 2/182 (1%)*
19	s208-Gene1-Lus10021711 class=Sequence position=scaffold208:731243..733997 (+ strand)	RecName: Full=Transcription factor bHLH30; AltName: Full=Basic helix-loop-helix protein 30; Short=AtbHLH30; Short=bHLH 30; AltName: Full=Transcription factor EN 53; AltName: Full=bHLH transcription factor bHLH030 Length=368	101/173 (58%)	1e-34	Q9S7Y1.1	GI:75336852	GO:0005634, GO:0003677, GO:0006355, GO:0006351,	*gb|EB713935.1|EB713935 LuP12022C09R LuP12 Linum usitatissimum cDNA clone LuP12022C09, mRNA sequence Length = 449 Score = 531 bits (268), Expect = e-149 Identities = 275/276 (99%), Gaps = 1/276 (0%)*
20	s208-Gene2-Lus10021712 class=Sequence position=scaffold208:741204..742074 (- strand)	No Hit						*gb|JG233667.1|JG233667 LUSTC1NG-RP-035_H07_27FEB2007_049 LUSTC1NG Linum usitatissimum cDNA, mRNA sequence Length = 626, Expect = 3e-034, Identities = 123/139 (88%)*
21	s305-Gene1-Lus10025162 class=Sequence position=scaffold305:591956..592495 (+ strand)	Full=RING-H2 finger protein ATL8 Length=185	74/117 (63%	8e-448	Q8LC69.2	GI:68565205	GO:0016021,GO:0008270, GO:0016567,	*gb|JG217382.1|JG217382 LUSPS1AD_RP_115_F02_18AUG2008_011 LUSPS1AD Linum usitatissimum cDNA, mRNA sequence Length = 905, Expect = 2e-030 Identities = 212/260 (81%)*
22	s305-Gene2-Lus10025163 class=Sequence position=scaffold305:596828..598803 (- strand)	Full=Probable inorganic phosphate transporter 1-9; Short=AtPht1;9; AltName: Full=H(+)/Pi cotransporter Length=532	161/307 (52%)	2e-77	Q9S735.1	GI:75313014	GO:0016021, GO:0015293, GO:0006817,	*LUSST3AD-T3-020_P17_5AUG2009_065, Length = 775, Expect = e-146, Identities = 263/263 (100%)*
23	s305-Gene3-Lus10025164 class=Sequence position=scaffold305:598835..600040 (+ strand)	Full=Probable inorganic phosphate transporter 1-8; Short=AtPht1;8; AltName: Full=H(+)/Pi cotransporter Length=534	78/128 (61%)	7e-40	Q9SYQ1.2	GI:85687566	GO:0016021, GO:0015293, GO:0006817	*LUSST3AD-T3-020_P17_5AUG2009_065, Length = 775, Expect = e-170,Identities = 306/307 (99%)*
24	s305-Gene4-Lus10025165 class=Sequence position=scaffold305:601003..603766 (- strand)	RecName: Full=SWI/SNF complex subunit SWI3B; Short=AtSWI3B; AltName: Full=Transcription regulatory protein SWI3B Length=469	137/271 (51%)	6e-74	Q84JG2.1	GI:75327834	GO:0005634, GO:0003677, GO:0016568, GO:0007275, GO:0006355, GO:0006351,	*gb|JG134955.1|JG134955 LUSHE1AD-RP-281_B14_3JUNE2008_063 LUSHE1AD Linum usitatissimum cDNA, mRNA sequence Length = 569 Score = 482 bits (243), Expect = e-134 Identities = 243/243 (100%)*
25	s225-Gene1-Lus10022341 class=Sequence position=scaffold225:786295..790333 (+ strand)	No Hit						*genolin_c24356 661 nt, Length = 3119, Expect = 0.0,Identities = 598/610 (98%)*
26	s225-Gene2-Lus10022342 class=Sequence position=scaffold225:790980..792191 (- strand)	Full=Probable adenylate kinase 1, chloroplastic; Short=AK 1; AltName: Full=ATP-AMP transphosphorylase 1; Flags: Precursor Length=284	56/151 (37%)	8e-19	Q9ZUU1.1	GI:29428074	GO:0005634, GO:0004017, GO:0005524, GO:0008652, GO:0048364, GO:0048367,	*gb|JG062715.1|JG062715 LUSES1AD_RP_103_F03_15JULY2008_012 LUSES1AD Linum usitatissimum,cDNA, mRNA sequence,Length = 729, Expect = 1e-021,Identities = 72/78 (92%)*

27	s225-Gene3-Lus10022343 class=Sequence position=scaffold225:800816..801259 (+ strand)	Full=Putative calcium-binding protein CML23; AltName: Full=Calmodulin-like protein 23 Length=151	81/150 (54%)	4e-42	Q8RYJ9.1	GI:75330796	GO:0005509	*genolin_c34622 211 nt,Length = 210, Expect = 5e-022, Identities = 54/54 (100%)*	
28	s225-Gene4-Lus10022344 class=Sequence position=scaffold225:802650..803564 (- strand)	Full=DnaJ homolog subfamily C member 21; AltName: Full=DnaJ homolog subfamily A member 5	68/219 (31%)	1e-18	Q6PGY5.1	GI:82187285	GO:0005622, GO:0003676, GO:0008270, GO:0006457,	*LUSGE1NG_RP_120_C12_09MAR2006_092.ab1,Length = 665, Expect = 0.0, Identities = 570/576 (98%)*	
29	s225-Gene5-Lus10022345 class=Sequence position=scaffold225:804542..806578 (- strand)	Full=Probable leucine-rich repeat receptor-like protein kinase At5g49770; Flags: Precursor Length=946	99/298 (33%)	2e-35	Q9LT96.1	GI:75335456	GO:0016021, GO:0005524,GO:0004674, GO:0004872,	*LUSGC1NG_RP_085_C09_24JAN2007_075.ab1,Length = 725, Expect = e-135,Identities = 332/360 (92%), Gaps = 7/360 (1%)*	
30	s86-gene1-Lus10040449 class=Sequence position=scaffold86:1969440..1978828 (- strand)	Full=Uncharacterized WD repeat-containing protein alr2800 Length=1258	50/207 (24%)	3e-11	Q8YTC2.1	GI:20140995	GO:0043531, GO:0006952,	*gb|JG214541.1|JG214541 LUSPS1AD_RP_106_D12_14AUG2008_046 LUSPS1AD Linum usitatissimum cDNA, mRNA sequence,Length = 913,Expect = e-134,Identities = 253/256 (98%)*	
31	s86-Gene2-Lus10040450 class=Sequence position=scaffold86:1979512..1979772 (+ strand)	No Hit	No Hits					*gb|JG285876.1|JG285876 LUSTE1NG-RP-179_C04_15FEB2007_028 LUSTE1NG Linum usitatissimum, cDNA, mRNA sequence,Length = 423, Expect = e-146, Identities = 261/261 (100%)*	
32	s86-Gene3-Lus10040451 class=Sequence position=scaffold86:1980748..1982274 (+ strand)	Full=H/ACA ribonucleoprotein complex subunit 4; AltName: Full=CBF5 homolog; AltName: Full=Dyskerin; AltName: Full=Nopp-140-associated protein of 57 kDa homolog; Short=AtNAP57; AltName: Full=Nucleolar protein NAP57 homolog Length=565	287/326 (88%),	0	Q9LD90.1	GI:67460428	GO:0005829,GO:0005730, GO:0009506, GO:0030529, GO:0009982, GO:0003723, GO:0001522, GO:0006364,	*gb|JG241371.1|JG241371 LUSTC1NG-RP-128_A01_07MAR2007_015 LUSTC1NG Linum usitatissimum cDNA, mRNA sequence, Length = 749, Expect = 0.0,Identities = 745/749 (99%)*	
33	s86-Gene4-Lus10040452 class=Sequence position=scaffold86:1983902..1984927 (- strand)	Putative F-box/LRR-repeat protein At5g02930 Length=469	45/159 (28%),	3e-9	Q9LYZ2.1	GI:75264447	No ontology	*gb|JG247257.1|JG247257 LUSTC1NG-RP-199_B06_17MAR2007_046 LUSTC1NG Linum usitatissimum,cDNA, mRNA sequence,Length = 633, Expect = 9e-004,Identities = 42/48 (87%)*	
34	s86-Gene5-Lus10040453 class=Sequence position=scaffold86:1987574..1987870 (- strand)	Full=DnaJ homolog subfamily C member 2; AltName: Full=M-phase phosphoprotein 11; AltName: Full=Zuotin-related factor 1 Length=621	23/52 (44%),	1e-5	Q99543.4	GI:296439472	GO:0005829, GO:0031965, GO:0003682,GO:0003677, GO:0051083, GO:0016568, GO:0006260, GO:0000085, GO:0030308, GO:0045893, GO:0006351,	*N:\Warehouse-Cloutier\Raja\flax-ESTs-NCBI-TUFGEN-FRENCH\All_flax-ESTs-NCBI-TUFGEN-FRENCH.txt 462,190 sequences; 259,282,616 total letters ***** No hits found *******	
35	s280-Gene1-Lus10041365 class=Sequence position=scaffold280:2125311..2128114 (+ strand)	No hit						gb|JG179335.1|JG179335 LUSLE4AD-T3-037_K22_06OCT2009_085 LUSLE1AD Linum usitatissimum cDNA, mRNA sequence, Length = 706, Expect = 0.0, Identities = 363/364 (99%)	

36	s280-Gene2-Lus10041366 class=Sequence position=scaffold280:2129130..2133045 (- strand)	RecName: Full=Probable methyltransferase PMT15 Length=633 GENE ID: 825923 AT4G00750 | putative methyltransferase PMT15	96/133 (72%)	5e-54	Q9ZPH9.1	GI:75267756	GO:0005794, GO:0000139, GO:0016021, GO:0008168,	*genolin_c39764 236 nt Length = 794, Expect = e-129 Identities = 236/236 (100%)*	
37	s280-Gene3-Lus10041367 class=Sequence position=scaffold280:2135272..2137021 (+ strand)	RecName: Full=F-box protein At4g00755 Length=377 GENE ID: 828014 AT4G00755 | F-box protein [Arabidopsis thaliana]	97/227	1e-42	Q8LG03.1	GI:75246091	No ontology	*gb|JG267291.1|JG267291 LUSTE1AD-RP-299_E04_27MAY2008_012 LUSTE1AD Linum usitatissimum cDNA,mRNA sequence, Expect = e-164, Identities = 293/293 (100%)*	
38	s280-Gene4-Lus10041368 class=Sequence position=scaffold280:2137745..2138988 (- strand)	RecName: Full=Peroxisomal membrane protein 11C; AltName: Full=Peroxin-11C; Short=AtPEX11c Length=235	153/361 (42%)	6e-65	Q9LQ73.1	GI:75180079	GO:0005779, GO:0009506,GO:0016559,	*LUSBE1NG_RP_056_C05_25OCT2006_043.ab1, Length = 674, Expect = e-128, Identities = 306/328 (93%), Gaps = 2/328 (0%)*	
39	s280-Gene5-Lus10041369 class=Sequence position=scaffold280:2140703..2142485 (- strand)	RecName: Full=Vacuolar protein 8 Length=556	33/133 (25%)	6e-7	Q5EFZ4.3	GI:74627608	GO:0005774, GO:0005488,	*gb|JG184357.1|JG184357 LUSME1AD-T3-011_J16_27JULY2009_056 LUSME1AD Linum usitatissimum, cDNA, mRNA sequence, Length = 908, Expect = 0.0,Identities = 619/620 (99%)*	
40	s280-Gene6-Lus10041370 class=Sequence position=scaffold280:2143673..2144155 (+ strand)	Full=Galactinol--sucrose galactosyltransferase; AltName: Full=Raffinose synthase Length=798	38/83 (46%),	5e-16	Q8VWN6.1	GI:75161213	GO:0047274, GO:0005975,	*genolin_c21095 396 nt, Length = 2251, Score = 228 bits (115), Expect = 2e-058, Identities = 220/253 (86%), Gaps = 9/253 (3%)*	
41	s280-Gene7-Lus10041371 class=Sequence position=scaffold280:2147074..2147469 (- strand)	No Hit						*Database: N:\Warehouse-Cloutier\Raja\flax-ESTs-NCBI-TUFGEN-FRENCH\All_flax-ESTs-NCBI-TUFGEN-FRENCH.txt, 462,190 sequences; 259,282,616 total letters, ***** No hits found *******	
42	scaffold98_Gene1_397284_397652	No hit						*gb|JG129297.1|JG129297 LUSGE1NG-RP-351_E04_11JAN2008_024 LUSGE1NG Linum usitatissimum, cDNA, mRNA sequence, Length =588, Expect = 0.0, Identities = 369/369 (100%)*	
43	scaffold98_Gene2_399369_399713	No Hit						*gb|JG041958.1|JG041958 03-LUSEN1NG-RP-042_H07_09MAR2007_049 LUSEN1NG Linum usitatissimum, cDNA, mRNA sequence, Length = 459,Score = 676 bits (341), Expect = 0.0, Identities = 344/345 (99%)*	expressed stem outer tissue (Fenart et al. 2010)
44	scaffold98_Gene3_402350_402766	Full=Glutaredoxin-C9 Length=192	69/110 (63%),	5e-29	Q7XIZ1.1	GI:75142699	GO:0005737, GO:0005634, GO:0009055, GO:0015035,GO:0045454, GO:0022900, GO:0006810,	*Database: N:\Warehouse-Cloutier\Raja\flax-ESTs-NCBI-TUFGEN-FRENCH\All_flax-ESTs-NCBI-TUFGEN-FRENCH.txt, 462,190 sequences; 259,282,616 total letters, ***** No hits found *******	

45	scaffold98_Gene4_406410_409511	Full=50S ribosomal protein L14	76/120 (63%)	1e-30	Q9ZCR5.1	GI:6225960	GO:0015934, GO:0019843, GO:0003735, GO:0006412,	*gb|JG019491.1|JG019491 03-LUSBE1NG-RP-022_E03_20OCT2006_023 LUSBE1NG Linum usitatissimum, cDNA, mRNA sequence,Length = 620, Expect = 0.0, Identities = 384/384 (100%)*
46	scaffold98_Gene5_414013_415454	RecName: Full=Transcription factor TCP2 Length=365	79/105 (75%),	4e-39	Q93V43.1	GI:75163104	GO:0005634, GO:0003677,GO:0030154, GO:0009965, GO:0045962, GO:0006355, GO:0006351,	*gb|JG226338.1|JG226338 LUSST4AD-T3-039_J14_15SEP2009_055 LUSST1AD Linum usitatissimum cDNA,mRNA sequence,Length = 900, Score = 1021 bits (515), Expect = 0.0,Identities = 524/527 (99%)*
47	scaffold98_Gene6_422900_424174	Full=F-box protein At5g46170 Length=395 GENE ID: 834659 AT5G46170 | F-box protein [Arabidopsis thaliana]	272/384 (71%)	4e-161	Q93V43.1	GI:75163104	GO:0005634, GO:0003677, GO:0030154, GO:0009965, GO:0045962, GO:0006355, GO:0006351,	*genolin_c34571 411 nt, Length = 2067, Score = 541 bits (273), Expect = e-152, Identities = 276/279 (98%)*
48	scaffold98_Gene7_425840_428477	Full=Probable ornithine aminotransferase; AltName: Full=Ornithine--oxo-acid aminotransferase Length=416	122/281 (43%)	2e-63	Q54JP5.1	GI:74896944	GO:0005737, GO:0004587, GO:0030170,GO:0006527, GO:0055129, GO:0006591,	*gb|JG108334.1|JG108334 LUSGE1NG-RP-080_A05_28FEB2007_047 LUSGE1NG Linum usitatissimum, cDNA, mRNA sequence,Length = 663, Expect = e-122, Identities = 233/236 (98%)*
49	scaffold98_Gene8_431313_435029	Full=KH domain-containing protein At4g18375 Length=606	109/200 (55%),	2e-52	P58223.1	GI:15214341	GO:0005634, GO:0003723,	*Genolin_c27804 465 nt similar to |AM435047|AM435047|Vitis vinifera contig, VV78X180421.8, whole genome shotgun sequence. Length = 465,Expect = 0.0,Identities = 389/405 (96%)*
50	scaffold98_Gene9_436341_437072	No hit						*gb|JG077706.1|JG077706 LUSFL2AD-WB-010_H20_22NOV2008_074 LUSFL1AD Linum usitatissimum,cDNA, mRNA sequence,Length = 390,Expect = e-161, Identities = 287/287 (100%)*
51	scaffold98_Gene10_438516_440980	Full=Peroxisomal (S)-2-hydroxy-acid oxidase; AltName: Full=Glycolate oxidase; Short=GOX; AltName: Full=Short chain alpha-hydroxy acid oxidase Length=369	83/165 (50%)	7e-71	P05414.1	GI:121530	GO:0005777, GO:0010181, GO:0052853, GO:0052854, GO:0052852, GO:0009854,	*gb|JG091780.1|JG091780 LUSGC1NG-RP-131_E08_30JAN2007_056 LUSGC1NG Linum usitatissimum cDNA,mRNA sequence, Length = 489,Expect = 2e-099, Identities = 191/193 (98%)*
52	scaffold98_Gene11_442330_442782	No hit						*gb|JG205486.1|JG205486 LUSME1NG-RP-189_A02_14APR2007_016 LUSME1NG Linum usitatissimum, cDNA, mRNA sequence, Length = 534,Expect = 0.0,Identities = 412/421 (97%)*

53	scaffold98_Gene12_446281_448331	RecName: Full=Pyruvate dehydrogenase E1 component subunit beta Length=326	250/322 (78%	4e-166	Q8MA03.1	GI:75272592	GO:0009507, GO:0004739, GO:0006096	*gb|JG256610.1|JG256610 LUSTE1AD-RP-266_D20_15MAY2008_078 LUSTE1AD Linum usitatissimum cDNA, mRNA sequence, Length = 792, Score = 1225 bits (618), Expect = 0.0,Identities = 621/622 (99%)*
54	scaffold98_Gene13_449529_450067	No hit						*gb|CA483034.1|CA483034 LuP12003H04R LuP12 Linum usitatissimum cDNA clone LuP12003H04R, mRNA sequence, Length = 742, Expect = e-166,Identities = 323/332 (97%)*
55	scaffold98_Gene14_450951_455179	RecName: Full=Cullin-4A; Short=CUL-4A Length=759	74/186 (40%),	1e-65	Q3TCH7.1	GI:108936014	GO:0031464, GO:0006281, GO:0044419, GO:0045732, GO:0045750, GO:0016567,GO:0006511	*gb|CA483034.1|CA483034 LuP12003H04R LuP12 Linum usitatissimum cDNA clone LuP12003H04R,mRNA sequence,Length = 742, Expect = e-137,Identities = 263/268 (98%)*
56	scaffold98_Gene15_456575_456808	No hit						*gb|JG192842.1|JG192842 LUSME1NG-RP-040_F01_21FEB2007_005 LUSME1NG Linum usitatissimum,cDNA, mRNA sequence, Length = 477,Expect = e-130,Identities = 234/234 (100%)*
57	scaffold98_Gene16_460382_460720	No Hit						*gb|JG088631.1|JG088631 LUSGC1NG-RP-094_D10_25JAN2007_074 LUSGC1NG Linum usitatissimum, cDNA, mRNA sequence, Length = 463, Expect = 0.004,Identities = 22/22 (100%)*
58	scaffold98_Gene17_465656_467484	Full=Glucan endo-1,3-beta-glucosidase 11; AltName: Full=(1->3)-beta-glucan endohydrolase 11; Short=(1->3)-beta-glucanase 11; AltName: Full=Beta-1,3-endoglucanase 11; Short=Beta-1,3-glucanase 11; Flags: Precursor Length=426	115/230 (50%),	3e-69	Q8L868.1	GI:75154301	GO:0046658,GO:0005618, GO:0005576, GO:0043169, GO:0042973, GO:0005975, GO:0007047, GO:0006952	*genolin_c42897 247 nt, Length = 862, Expect = e-128, Identities = 243/245 (99%), Gaps = 1/245 (0%)*
59	scaffold98_Gene18_470801_476488	RecName: Full=Katanin p60 ATPase-containing subunit A-like 2; Short=Katanin p60 subunit A-like 2; AltName: Full=p60 katanin-like 2 Length=538	67/132 (51%)	5e-27	Q8IYT4.3	GI:189028467	GO:0005737, GO:0005874, GO:0005524, GO:0008568,	*gb|JG130726.1|JG130726 LUSGE1NG-RP-368_A06_17JAN2007_048 LUSGE1NG Linum usitatissimum cDNA, mRNA sequence, Length = 754, Expect = 0.0, Identities = 396/396 (100%)*
60	scaffold98_Gene19_488842_490064	Full=Gibberellin 2-beta-dioxygenase; AltName: Full=GA 2-oxidase; AltName: Full=Gibberellin 2-beta-hydroxylase; AltName: Full=Gibberellin 2-oxidase Length=332	72/116 (62%)	7e-108	Q9XG83.1	GI:49035968	GO:0045543, GO:0005506, GO:0016702, GO:0009686,	*genolin_c40872 256 nt, Length = 2190, Expect = 8e-097, Identities = 180/180 (100%)*

61	scaffold98_Gene20_499753_503369	No Hit						*gb|JG053799.1|JG053799 03-LUSEN1NG-RP-179_H03_28MAR2007_017 LUSEN1NG Linum usitatissimum, cDNA, mRNA sequence, Length = 648, Expect = e-180, Identities = 411/441 (93%)*	expressed stem inner tissue (Fenart et al. 2010)
62	scaffold98_Gene21_506403_508325	RecName: Full=Probable prefoldin subunit 6 Length=125	28/44 (64%)	1e-7	Q9VW56.1	GI:12230499	GO:0016272, GO:0006457,	*gb|JG281587.1|JG281587 LUSTE1NG-RP-130_H01_6FEB2006_001 LUSTE1NG Linum usitatissimum cDNA, mRNA sequence,Length = 671, Expect = 0.0,Identities = 667/671 (99%)*	
63	scaffold98_Gene22_509753_513251	No hit						*LUSHE1AD-RP-277_O04_30MAY2008_002, Length = 781, Expect = 0.0,Identities = 611/611 (100%)*	
64	scaffold98_Gene23_513885_516730	No hit						*gb|JG207296.1|JG207296 LUSME1NG-RP-209_C05_28NOV2007_043 LUSME1NG Linum usitatissimum, cDNA, mRNA sequence, Length = 587, Expect = 1e-085, Identities = 201/214 (93%)*	
65	scaffold98_Gene24_518171_520057	Full=Phospho-N-acetylmuramoyl-pentapeptide-transferase homolog; AltName: Full=Translocase I Length=480	102/182 (56%)	1e-36	O49730.3	GI:229621258	GO:0016021, GO:0008963,	*gb|JG244959.1|JG244959 LUSTC1NG-RP-171_G04_17MAR2007_020 LUSTC1NG Linum usitatissimum cDNA,mRNA sequence,Length = 685,Expect = 8e-092, Identities = 202/212 (95%)*	
66	scaffold98_Gene25_525066_525488	Full=Probable purine permease 10; Short=AtPUP10 Length=390	73/126 (58%),	1e-42	O49725.2	GI:167012003	GO:0016021, GO:0016020, GO:0005345, GO:0009624,	*Database: N:\Warehouse-Cloutier\Raja\flax-ESTs-NCBI-TUFGEN-FRENCH\All_flax-ESTs-NCBI-TUFGEN-FRENCH.txt, 462,190 sequences; 259,282,616 total letters, ***** No hits found *******	
67	scaffold98_Gene26_525551_526798	scaffold98_Gene26_525551_526798	51/81 (63%),	7e-21	O49725.2	GI:167012003	GO:0016021, GO:0016020, GO:0005345, GO:0009624,	*genolin_c13306 513 nt,Length = 511, Expect = 0.0, Identities = 328/328 (100%)*	
68	scaffold98_Gene27_528586_533646	Full=Glycogen synthase 2; AltName: Full=Starch [bacterial glycogen] synthase 2 Length=487	66/159 (42%)	2e-30	Q604D9.2	GI:91206712	GO:0009011, GO:0005978,	*genolin_c30229 306 nt, Length = 1022, Expect = 0.0,Identities = 671/676 (99%)*	
69	scaffold98_Gene28_533921_534217	Full=Chlorophyll a-b binding protein 1D; AltName: Full=LHCII type I CAB-1D; Short=LHCP Length=116	92/98 (94%),	1e-64	P10707.1	GI:115822	GO:0009535, GO:0016021, GO:0009522, GO:0009523, GO:0016168, GO:0046872, GO:0009765, GO:0018298,	*gb|JG222986.1|JG222986 LUSST4AD-T3-020_O01_15SEP2009_001 LUSST1AD Linum usitatissimum, cDNA, mRNA sequence, Length = 744*	

70	scaffold98_Gene29_534348_534719	Chlorophyll a-b binding protein 16, chloroplastic; AltName: Full=LHCII type I CAB-16; Short=LHCP; Flags: Precursor Length=266	82/103 (80%	2e-54	P27492.1	GI:115781	GO:0009535, GO:0016021, GO:0009522, GO:0009523,GO:0016168, GO:0046872, GO:0009765, GO:0018298,	*gb|JG212961.1|JG212961 LUSPS1AD_RP_101_H13_11AUG2008_057 LUSPS1AD Linum usitatissimum,cDNA, mRNA sequence,Length = 868,Expect = 0.0,Identities = 362/372 (97%), Gaps = 1/372 (0%)*
71	scaffold98_Gene30_536533_537144	Full=Chlorophyll a-b binding protein 3C, chloroplastic; AltName: Full=LHCII type I CAB-3C; Short=LHCP; Flags: Precursor Length=267	178/266 (67%)	1e-114	P07369.1	GI:115825	GO:0009535, GO:0016021, GO:0009522, GO:0009523, GO:0016168, GO:0046872, GO:0009765, GO:0018298,	*gb|JG222967.1|JG222967 LUSST4AD-T3-020_M07_15SEP2009_020 LUSST1AD Linum usitatissimum,cDNA, mRNA sequence,Length = 819, Expect = 0.0,Identities = 365/365 (100%)*
72	scaffold98_Gene31_537930_542065	Full=LRR receptor-like serine/threonine-protein kinase FLS2; AltName: Full=Protein FLAGELLIN-SENSING 2; AltName: Full=Protein FLAGELLIN-SENSITIVE 2; Flags: Precursor Length=1173	473/945 (50%)	0	Q9FL28.1	GI:75262640	GO:0010008, GO:0016021, GO:0005886, GO:0005524, GO:0004674, GO:0004872, GO:0052544, GO:0042742, GO:0016045, GO:0010359,	*genolin_c28812 266 nt, Length = 1422,Expect = e-140, Identities = 263/265 (99%), Gaps = 1/265 (0%)*
73	scaffold98_Gene32_544581_544823	RecName: Full=Probable receptor-like protein kinase At5g38990; Flags: Precursor Length=880	25/70 (36%)	3e-06	Q9FID9.1	GI:75333907	GO:0016021, GO:0005524, GO:0004674,	*genolin_c12301 193 nt, Length = 3746, Expect = 1e-008,Identities = 58/67 (86%)*
74	scaffold98_Gene33_545289_545978	Receptor-like protein kinase HERK 1; AltName: Full=Protein HERCULES RECEPTOR KINASE 1; Flags: Precursor Length=830	82/225 (36%)	2e-25	Q9LX66.1	GI:75335601	GO:0016021, GO:0005886, GO:0009506, GO:0005524, GO:0004672,GO:0004674, GO:0004872, GO:0009742, GO:0009791, GO:0051510,GO:0009826,	*genolin_c12301 193 nt, Length = 3746, Expect = 4e-008, Identities = 34/35 (97%)*
75	scaffold98_Gene34_546962_548755	Full=Polyneuridine-aldehyde esterase; AltName: Full=Polyneuridine aldehyde esterase; Flags: Precursor Length=264	57/108 (53%),	1e-28	Q9SE93.1	GI:50401192	GO:0004091, GO:0050529, GO:0009820	*gb|JG225691.1|JG225691 LUSST4AD-T3-036_C21_15SEP2009_093 LUSST1AD Linum usitatissimum cDNA, mRNA sequence, Length = 809, Expect = e-167, Identities = 298/298 (100%)*
76	scaffold98_Gene35_549762_550229	No hit						*gb|JG101512.1|JG101512 LUSGC1NG-RP-245_F08_02APR2007_054 LUSGC1NG Linum usitatissimum, cDNA, mRNA sequence, Length = 563, Score = 77.8 bits (39), Expect = 4e-013, Identities = 54/59 (91%)*
77	scaffold98_Gene36_553003_560612	Full=Calcium-transporting ATPase 1, endoplasmic reticulum-type Length=1061	388/467 (83%)	0	P92939.2	GI:12643704	GO:0030176,GO:0005886, GO:0005774, GO:0005524, GO:0005388, GO:0046872, GO:0030026, GO:0006828, GO:0046686, GO:0010042	*genolin_c16266 441 nt,Length = 1851, Expect = 0.0,Identities = 736/758 (97%), Gaps = 3/758 (0%)*

78	scaffold98_Gene37_561231_562778	Full=TMV resistance protein N Length=1144	56/182 (31%),	5e-28	Q40392.1	GI:46577339	GO:0005737, GO:0043531, GO:0005524, GO:0009626, GO:0007165	*gb|JG250981.1|JG250981 LUSTC1NG-RP-243_D11_15MAY2007_089 LUSTC1NG Linum usitatissimum cDNA, mRNA sequence Length = 661, Expect = 0.001,Identities = 33/36 (91%)*	
79	scaffold98_Gene38_563086_563918	RecName: Full=Leucine-rich repeat-containing protein 40 Length=602	36/120 (30%)	4e-8	Q9H9A6.1	GI:74761553	No ontology	***** No hits found ******	
80	scaffold98_Gene39_566883_567194	Full=Histone H4 variant TH091 Length=103	82/82 (100%)	5e-51	P62786.2	GI:51338727	GO:0000786, GO:0005634, GO:0003677,GO:0006334,	*gb|JG130751.1|JG130751 LUSGE1NG-RP-368_C11_17JAN2007_091 LUSGE1NG Linum usitatissimum, cDNA, mRNA sequence Length = 614,Score = 618 bits (312), Expect = e-176,Identities = 312/312 (100%)*	
81	scaffold98_Gene40_572911_573630	Full=WUSCHEL-related homeobox 3; AltName: Full=Protein PRESSED FLOWER Length=244	47/57 (82%)	3e-25	Q9SIB4.1	GI:61217434	GO:0005634, GO:0043565, GO:0003700,GO:0009943, GO:0030154, GO:0008283, GO:0009947, GO:0009908,GO:0010865, GO:0006351,	*gb|JG214412.1|JG214412 LUSPS1AD_RP_105_N07_14AUG2008_020 LUSPS1AD Linum usitatissimum,cDNA, mRNA sequence Length = 898, Expect = 6e-010,Identities = 49/54 (90%)*	
82	scaffold98_Gene41_579841_580740	Full=Chitinase 1; AltName: Full=Tulip bulb chitinase-1; Short=TBC-1; Flags: Precursor Length=314	165/278 (59%)	1e-112	Q9SLP4.1	GI:47605559	GO:0043169, GO:0008061, GO:0004568, GO:0006032,	*gb|JG106882.1|JG106882 LUSGE1NG-RP-063_C11_23FEB2007_091 LUSGE1NG Linum usitatissimum,cDNA, mRNA sequence Length = 692,Expect = 0.0,Identities = 325/326 (99%)*	
83	scaffold98_Gene42_582604_583287	No hit					No Hit	*LUSGE1NG_RP_191_C10_04APR2007_076.ab1, Length = 727, Expect = 0.0,Identities = 561/561 (100%)*	
84	scaffold98_Gene43_583631_584381	Full=Receptor-like serine/threonine-protein kinase SD1-8; AltName: Full=Arabidopsis thaliana receptor kinase 3; AltName: Full=S-domain-1 (SD1) receptor kinase 8; Short=SD1-8; Flags: Precursor Length=850	58/140 (41%),	8e-34	O81905.1	GI:75318808	GO:0016021, GO:0005886, GO:0009506, GO:0005773, GO:0005524,GO:0004674, GO:0004872, GO:0048544,	*genolin_c35587 636 nt,Length = 635,Score = 165 bits (83), Expect = 4e-039,Identities = 119/131 (90%)*	
85	scaffold98_Gene44_585760_588145	RecName: Full=BTB/POZ domain-containing protein At5g60050 Length=499	193/322 (60%),	3e-110	Q9LVG9.1	GI:75180651	No ontology	*gb|EH792489.1|EH792489 LU01UID.9374 stem phloem (bast) fiber enriched library LU01 Linum usitatissimum cDNA clone FLAXPH19_UP_001_D05, mRNA sequence Length = 1246 Expect = 0.0 dentities = 799/820 (97%), Gaps = 12/820 (1%)*	expressed stem outer tissue (Fenart et al. 2010)

86	scaffold98_Gene45_589675_592030	Full=ATP synthase subunit d, mitochondrial; Short=ATPase subunit d Length=168	45/57 (79%),	9e-22	Q9FT52.3	GI:25089786	GO:0009535, GO:0022626, GO:0005753, GO:0000276, GO:0005730, GO:0005774, GO:0005507, GO:0015078,GO:0016787, GO:0008270, GO:0015986, GO:0009651,	*gb|JG266257.1|JG266257 LUSTE1AD-RP-296_D04_26MAY2008_014 LUSTE1AD Linum usitatissimum cDNA,mRNA sequence Length = 846, Expect = 2e-092,Identities = 173/173 (100%)*
87	scaffold98_Gene46_593212_595545	No Hit						*gb|JG227288.1|JG227288 LUSST4AD-T3-044_N02_15SEP2009_003 LUSST1AD Linum usitatissimum cDNA,mRNA sequence Length = 914,Expect = e-172,Identities = 343/355 (96%)*
88	scaffold98_Gene47_598919_602248	Full=Mitogen-activated protein kinase 3; Short=AtMPK3; Short=MAP kinase 3 Length=370	97/114 (85%)	2e-57	Q39023.2	GI:21431794	GO:0005737,GO:0005634, GO:0005524, GO:0004707, GO:0004672, GO:0009738,GO:0000169, GO:0010120, GO:0000165, GO:0048481, GO:0009626, GO:0080136, GO:2000038, GO:2000037, GO:0009617, GO:0010200,GO:0009409, GO:0006970, GO:0006979, GO:0010224, GO:0009611,	*gb|JG143087.1|JG143087 LUSHE1AD-RP-307_M16_16JUN2008_052 LUSHE1AD Linum usitatissimum cDNA,mRNA sequence,Length = 767, Expect = 2e-010,Identities = 87/104 (83%)*
89	scaffold98_Gene48_604947_610070	Full=Non-lysosomal glucosylceramidase; Short=NLGase; AltName: Full=Beta-glucocerebrosidase 2; Short=Beta-glucosidase 2; AltName: Full=Glucosylceramidase 2 Length=927	66/117 (56%)	2e-29	Q9HCG7.2	GI:143018392	GO:0016021,GO:0005792, GO:0005886, GO:0005790, GO:0008422, GO:0004348, GO:0008206, GO:0006680, GO:0016139, GO:0006687,	*gb|JG240063.1|JG240063 LUSTC1NG-RP-112_D08_06MAR2007_058 LUSTC1NG Linum usitatissimum cDNA,mRNA sequence,Length = 704, Expect = e-153,Identities = 278/279 (99%)*
90	scaffold98_Gene49_611633_615677	RecName: Full=DNA repair protein RAD51 homolog 2; Short=AtRAD51B Length=370	78/173 (45%)	1e-19	Q9SK02.2	GI:83305358	GO:0005634, GO:0005524, GO:0003677, GO:0008094, GO:0006310, GO:0006281,	*Database: N:\Warehouse-Cloutier\Raja\flax-ESTs-NCBI-TUFGEN-FRENCH\All_flax-ESTs-NCBI-TUFGEN-FRENCH.txt,462,190 sequences; 259,282,616 total letters,***** No hits found *******
91	scaffold98_Gene50_616904_617501	No hit						*gb|JG124790.1|JG124790 LUSGE1NG-RP-299_B02_23NOV2007_014 LUSGE1NG Linum usitatissimum, cDNA, mRNA sequence, Length = 498,Expect = e-104,Identities = 207/212 (97%)*
92	scaffold98_Gene51_617938_619130	No hit						*genolin_c17438 413 nt, Length = 1641, Expect = 4e-006,Identities = 31/32 (96%)*

93	scaffold98_Gene52_621335_628038	Full=E3 SUMO-protein ligase SIZ1 Length=884	163/367 (44%)	2e-89	Q680Q4.2	GI:73919315	GO:0016607,GO:0005634, GO:0003676, GO:0019789, GO:0008270, GO:0051301, GO:0016049, GO:0016036, GO:0006952, GO:0010247, GO:0048589,GO:0009908, GO:0010286, GO:0009910, GO:0010113, GO:0016925, GO:0009787, GO:0040008, GO:0090352, GO:2000070, GO:0050826, GO:0009414,GO:0010337,	*genolin_c41078 239 nt,Length = 239, Expect = e-125,Identities = 235/237 (99%)*
94	scaffold98_Gene53_628666_637849	No hit						*gb|JG219065.1|JG219065 LUSST3AD-T3-013_F09_5AUG2009_043 LUSST1AD Linum usitatissimum cDNA,mRNA sequence,Length = 744,Expect = 0.0,Identities = 737/741 (99%)*
95	scaffold98_Gene54_639158_643411	Full=Calcium-dependent protein kinase 17 Length=528	160/172 (93%),	5e-99	Q9FMP5.1	GI:75334077	GO:0005737, GO:0005886, GO:0005524,GO:0005509, GO:0004674, GO:0046777, GO:0080092,	*gb|JG074943.1|JG074943 LUSFL1AD-WB-009_H11_09NOV2008_042 LUSFL1AD Linum usitatissimum cDNA,mRNA sequence Length = 934,Expect = 9e-081,Identities = 154/154 (100%)*
96	scaffold98_Gene55_645707_646558	No hit						*gb|JG189528.1|JG189528 LUSME2AD-T3-023_B10_6AUG2009_047 LUSME1AD Linum usitatissimum cDNA,mRNA sequence,Length = 766, Expect = 2e-004,Identities = 28/29 (96%)*
97	scaffold98_Gene56_649787_652129	Full=Tubulin beta-5 chain; AltName: Full=Beta-5-tubulin Length=447	206/210 (98%)	2e-139	P46265.1	GI:1174600	GO:0005618, GO:0009507, GO:0005874,GO:0005886, GO:0005525, GO:0003924, GO:0005198, GO:0007018, GO:0051258, GO:0046686,	*gb|JG075806.1|JG075806 LUSFL1AD-WB-024_I20_10NOV2008_072 LUSFL1AD Linum usitatissimum cDNA,mRNA sequence,Length = 924,Expect = 0.0,Identities = 664/666 (99%)*
98	scaffold98_Gene57_652941_653766	Full=Putative germin-like protein 2-1; Flags: Precursor Length=216	85/176 (48%)	7e-54	Q6K5Q0.1	GI:75261355	GO:0048046, GO:0005618,GO:0031012, GO:0030145, GO:0045735, GO:0009651,	*gb|JG232323.1|JG232323 LUSTC1NG-RP-020_A05_26FEB2007_047 LUSTC1NG Linum usitatissimum,cDNA, mRNA sequence Length = 579, Expect = 4e-073,Identities = 225/252 (89%), Gaps = 1/252 (0%)*
99	scaffold98_Gene58_655609_656369	Full=Putative germin-like protein 2-1; Flags: Precursor Length=216	81/175 (46%	4e-52	Q6K5Q0.1	GI:75261355	GO:0048046, GO:0005618,GO:0031012, GO:0030145, GO:0045735, GO:0009651,	*gb|JG232323.1|JG232323 LUSTC1NG-RP-020_A05_26FEB2007_047 LUSTC1NG Linum usitatissimum,cDNA, mRNA sequence Length = 579,Expect = e-103,Identities = 240/255 (94%), Gaps = 1/255 (0%)*

100	scaffold98_Gene59_660073_660723	Full=Putative germin-like protein 2-1; Flags: Precursor Length=216	131/216 (61%)	2e-88	Q6K5Q0.1	GI:75261355	GO:0048046, GO:0005618,GO:0031012, GO:0030145, GO:0045735, GO:0009651,	*gb|JG214720.1|JG214720 LUSPS1AD_RP_106_M16_14AUG2008_052 LUSPS1AD Linum usitatissimum,cDNA, mRNA sequence,Length = 879,Score = 56.0 bits (28), Expect = 2e-006,Identities = 34/36 (94%)*	
101	scaffold98_Gene60_661505_663492	Full=Probable protein phosphatase 2C 28; Short=AtPP2C28 Length=339	23/35 (66%)	8e-6	O64583.2	GI:391358160	GO:0046872,GO:0004721, GO:0008152,	*gb|JG193905.1|JG193905 LUSME1NG-RP-052_B05_21FEB2007_045 LUSME1NG Linum usitatissimum, cDNA, mRNA sequence, Length = 531, Expect = 2e-061,Identities = 145/153 (94%)*	expressed stem inner tissue (Fenart et al. 2010)
102	scaffold98_Gene61_664643_671706	Full=PHD finger-containing protein DDB_G0268158 Length=688	50/165 (30%)	2e-6	Q55FD6.1	GI:74859221	GO:0008270,	*b|JG224385.1|JG224385 LUSST4AD-T3-028_I01_15SEP2009_007 LUSST1AD Linum usitatissimum,cDNA, mRNA sequence,Length = 888, Expect = 0.0,Identities = 708/709 (99%)*	expressed stem inner tissue (Fenart et al. 2010)
103	scaffold98_Gene62_673162_680563	No hit						*genolin_c11483 690 nt,Length = 2315, Expect = 0.0, Identities = 476/479 (99%)*	expressed stem inner tissue (Fenart et al. 2010)
104	scaffold98_Gene63_685661_687664	Full=DNA-damage-repair/toleration protein DRT111, chloroplastic; Flags: Precursor Length=387	186/292 (64%)	2e-58	P42698.2	GI:20141383	GO:0009507, GO:0005737, GO:0005634, GO:0000166, GO:0003723, GO:0006281,	*genolin_c36779 288 nt,Length = 288, Score = 93.7 bits (47), Expect = 3e-017, Identities = 116/139 (83%)*	
105	scaffold98_Gene64_688645_689527	No hit						*gb|JG132151.1|JG132151 LUSHE1AD-RP-272_I07_30MAY2008_024 LUSHE1AD Linum usitatissimum, cDNA, mRNA sequence, Length = 802,Expect = 8e-081,Identities = 153/153 (100%)*	
106	scaffold98_Gene65_692597_697218	Full=Homeobox-leucine zipper protein HOX32; AltName: Full=HD-ZIP protein HOX32; AltName: Full=Homeodomain transcription factor HOX32; AltName: Full=OsHox32 Length=859	134/245 (55%)	0	Q6AST1.1	GI:75119691	GO:0005634, GO:0043565, GO:0003700, GO:0006351,	*gb|JG179263.1|JG179263 LUSLE4AD-T3-037_F16_06OCT2009_060 LUSLE1AD Linum usitatissimum, cDNA, mRNA sequence, Length = 931, Score = 660 bits (333), Expect = 0.0, Identities = 333/333 (100%)*	
107	scaffold98_Gene66_699934_700982	Full=Nuclear transcription factor Y subunit A-7; Short=AtNF-YA-7 Length=190	48/58 (83%)	2e-23	Q84JP1.1	GI:75146690	GO:0005634, GO:0003677, GO:0003700,GO:0045892, GO:0006351,	*gb|JG214921.1|JG214921 LUSPS1AD_RP_107_G14_15AUG2008_057 LUSPS1AD Linum usitatissimum, cDNA, mRNA sequence,Length = 890, Expect = e-106,Identities = 196/196 (100%)*	
108	scaffold98_Gene67_705511_706715	Full=Fatty acid 2-hydroxylase; AltName: Full=Fatty acid alpha-hydroxylase Length=372	47/143 (33%),	7e-36	Q2LAM0.2	GI:162416308	GO:0005789, GO:0016021, GO:0005792, GO:0020037, GO:0016491,GO:0022900, GO:0006633, GO:0006665, GO:0006810,	*gb|JG265935.1|JG265935 LUSTE1AD-RP-295_E05_26MAY2008_027 LUSTE1AD Linum usitatissimum, cDNA, mRNA sequence,Length = 723, Expect = 3e-078,Identities = 149/149 (100%)*	

109	scaffold98_Gene68_708381_714139	Full=Myosin-2 heavy chain; AltName: Full=Myosin II heavy chain Length=2116	106/479 (22%)	7e-11	P08799.3	GI:134047850	GO:0042641, GO:0005826, GO:0032009, GO:0032982, GO:0016460, GO:0001931, GO:0030898, GO:0005524, GO:0000146, GO:0033275, GO:0032060, GO:0006935, GO:0030038, GO:0030866, GO:0031154, GO:0000910,GO:0060328, GO:0046847, GO:0031034, GO:0030837, GO:0008104,GO:0031270, GO:0034461	*LUSPS1AD_RP_104_H24_14AUG2008_090, Length = 919,Expect = e-173,Identities = 310/310 (100%)*	expressed stem outer tissue (Fenart et al. 2010)
110	scaffold98_Gene69_723202_724905	Full=Reticuline oxidase-like protein; Flags: Precursor Length=570	227/538 (42%)	1e-135	Q9SVG4.2	GI:118585329	GO:0031225, GO:0048046,GO:0005829, GO:0005739, GO:0009505, GO:0005886, GO:0009506,GO:0005773, GO:0050660, GO:0008762, GO:0006979,	*LUSTC1NG_RP_153_B08_12MAR2007_062.ab1, Length = 715, Expect = 0.0, Identities = 549/549 (100%)*	
111	scaffold98_Gene70_735327_736578	No hit						*genolin_c19393 422 nt,Length = 1037, Expect = 0.004,Identities = 53/63 (84%)*	
112	scaffold98_Gene71_736742_738728	Full=Reticuline oxidase-like protein; Flags: Precursor Length=570	165/361 (46%)	6e-90	Q9SVG4.2	GI:118585329	GO:0031225, GO:0048046, GO:0005829, GO:0005739, GO:0009505, GO:0005886, GO:0009506,GO:0005773, GO:0050660, GO:0008762, GO:0006979	*genolin_c35055 364 nt, Length = 1078, Expect = 7e-009,Identities = 39/41 (95%)*	
113	scaffold98_Gene72_741160_741579	Full=Polygalacturonase; Short=PG; AltName: Full=Pectinase; Flags: Precursor Length=396	63/113 (56%),	8e-35	Q05967.1	GI:548491	GO:0005618, GO:0005576, GO:0004650, GO:0005975,GO:0007047	*gb|JG181357.1|JG181357 LUSLE4AD-T3-052_I23_13NOV2009_088 LUSLE1AD Linum usitatissimum, cDNA, mRNA sequence, Length = 934, Expect = 0.005,Identities = 37/42 (88%)*	
114	scaffold98_Gene73_741731_743788	Full=Reticuline oxidase-like protein; Flags: Precursor Length=570	200/443 (45%)	3e-103	Q9SVG4.2	GI:118585329	GO:0031225, GO:0048046,GO:0005829, GO:0005739, GO:0009505, GO:0005886, GO:0009506, GO:0005773, GO:0050660, GO:0008762, GO:0006979,	*genolin_c21177 543 nt, Length = 1581,Expect = 3e-005,Identities = 99/123 (80%)*	
115	scaffold98_Gene74_745613_746839	Full=Reticuline oxidase-like protein; Flags: Precursor Length=570	248/407 (61%)	1e-159	Q9SVG4.2	GI:118585329	GO:0031225, GO:0048046,GO:0005829, GO:0005739, GO:0009505, GO:0005886, GO:0009506,GO:0005773, GO:0050660, GO:0008762, GO:0006979,	*genolin_c16532 283 nt, Length = 895, Expect = 0.0,Identities = 539/558 (96%), Gaps = 3/558 (0%)*	

116	scaffold98_Gene75_749340_749810	Full=Reticuline oxidase-like protein; Flags: Precursor Length=570	71/155 (46%)	1e-37	Q9SVG4.2	GI:118585329	GO:0031225, GO:0048046, GO:0005829, GO:0005739, GO:0009505, GO:0005886, GO:0009506, GO:0005773, GO:0050660, GO:0008762, GO:0006979,	*Database: N:\Warehouse-Cloutier\Raja\flax-ESTs-NCBI-TUFGEN-FRENCH\All_flax-ESTs-NCBI-TUFGEN-FRENCH.txt, 462,190 sequences; 259,282,616 total letters, ***** No hits found *******
117	scaffold98_Gene76_750256_751770	Full=Reticuline oxidase-like protein; Flags: Precursor Length=570	62/92 (67%),	5e-34	Q9SVG4.2	GI:118585329	GO:0031225, GO:0048046,GO:0005829, GO:0005739, GO:0009505, GO:0005886, GO:0009506,GO:0005773, GO:0050660, GO:0008762, GO:0006979	*gb|JG284709.1|JG284709 LUSTE1NG-RP-166_A10_10FEB2007_080 LUSTE1NG Linum usitatissimum, cDNA, mRNA sequence, Length = 747, Score = 163 bits (82), Expect = 3e-038,Identities = 142/162 (87%)*
118	scaffold98_Gene77_751882_752268	Full=Reticuline oxidase-like protein; Flags: Precursor Length=570	58/122 (48%)	2e-32	Q9SVG4.2	GI:118585329	GO:0031225, GO:0048046, GO:0005829, GO:0005739, GO:0009505, GO:0005886, GO:0009506,GO:0005773, GO:0050660, GO:0008762, GO:0006979,	*genolin_c40702 227 nt, Length = 1428, Expect = 8e-005, Identities = 37/41 (90%)*
119	scaffold98_Gene78_752452_753635	No hit						*genolin_c21859 375 nt,Length = 1420, Expect = e-161,Identities = 300/304 (98%)*
120	scaffold98_Gene79_756010 756804 + . ID=Lus10023371; (+ strand)	Full=Intracellular ribonuclease LX; Short=RNase LX; Flags: Precursor Length=237	54/217 (25%)	2e-11	P80196.2	GI:1710616	GO:0005737, GO:0033897, GO:0003723, GO:0090305,GO:0006950,	*Database: N:\Warehouse-Cloutier\Raja\flax-ESTs-NCBI-TUFGEN-FRENCH\All_flax-ESTs-NCBI-TUFGEN-FRENCH.txt, 462,190 sequences; 259,282,616 total letters, ***** No hits found *******
121	scaffold98_Gene80_757680_759425	Full=Putative F-box protein At3g16210 Length=360	52/179 (29%)	4e-10	Q9LU24.1	GI:75274170	No ontology	*gb|EB711981.1|EB711981 LuP12012F11R LuP12 Linum usitatissimum cDNA clone LuP12012F11, mRNA sequence, Length = 407, Expect = 0.0,Identities = 397/407 (97%)*
122	scaffold98_Gene81_759609_760532	Full=Reticuline oxidase-like protein; Flags: Precursor Length=570	118/289 (41%)	2e-72	Q9SVG4.2	GI:118585329	GO:0031225, GO:0048046, GO:0005829, GO:0005739, GO:0009505, GO:0005886, GO:0009506,GO:0005773, GO:0050660, GO:0008762, GO:0006979	*genolin_c40702 227 nt, Length = 1428, Expect = 2e-004,Identities = 37/41 (90%)*
123	scaffold98_Gene82_762275_763798	Full=Reticuline oxidase-like protein; Flags: Precursor Length=570	224/507 (44%	3e-131	Q9SVG4.2	GI:118585329	GO:0031225, GO:0048046,GO:0005829, GO:0005739, GO:0009505, GO:0005886, GO:0009506, GO:0005773, GO:0050660, GO:0008762, GO:0006979,	*gb|JG242658.1|JG242658 LUSTC1NG-RP-143_F11_11MAR2007_085 LUSTC1NG Linum usitatissimum, cDNA, mRNA sequence, Length = 548, Expect = 3e-072,Identities = 319/379 (84%)*

124	scaffold98_Gene83_769673_771286	Full=Reticuline oxidase-like protein; Flags: Precursor Length=570	271/511 (53%)	1e-154	Q9SVG4.2	GI:118585329	GO:0031225, GO:0048046, GO:0005829, GO:0005739, GO:0009505, GO:0005886, GO:0009506,GO:0005773, GO:0050660, GO:0008762, GO:0006979,	*genolin_c35272 350 nt, Length = 773, Expect = 0.0,Identities = 363/363 (100%)*
125	scaffold98_Gene84_774850_775857	Full=Reticuline oxidase-like protein; Flags: Precursor Length=570	118/355 (33%)	3e-48	Q9SVG4.2	GI:118585329	GO:0031225, GO:0048046, GO:0005829, GO:0005739, GO:0009505, GO:0005886, GO:0009506,GO:0005773, GO:0050660, GO:0008762, GO:0006979,	*genolin_c40702 227 nt, Length = 1428, Expect = 1e-011,Identities = 82/97 (84%)*
126	scaffold98_Gene85_776136_776447	RecName: Full=Patellin-4 Length=540	60/99 (61%)	2e-35	Q94C59.2	GI:78099068	GO:0005829, GO:0016021, GO:0005634, GO:0005886, GO:0008289, GO:0005215, GO:0007049, GO:0051301,	*LUSTC1NG_RP_011_G02_26FEB2007_004.ab1, Length = 681, Expect = e-174,Identities = 311/312 (99%)*
127	scaffold98_Gene86_776618_777622	Full=Patellin-4 Length=540	158/335 (47%)	4e-76	Q94C59.2	GI:78099068	GO:0005829, GO:0016021, GO:0005634, GO:0005886, GO:0008289, GO:0005215, GO:0007049, GO:0051301,	*genolin_c28034 619 nt, Length = 1092, Expect = 0.0, Identities = 428/450 (95%), Gaps = 12/450 (2%)*
128	scaffold98_Gene87_780614_786266	No hit						*genolin_c32131 396 nt, Length = 644, Expect = 2e-095,Identities = 180/181 (99%)*
129	scaffold98_Gene88_787152_789714	No hit						*genolin_c24422 395 nt, Length = 3305,Expect = 2e-077,Identities = 160/164 (97%)*
130	scaffold98_Gene89_806547_811420	Full=WD40 repeat-containing protein SMU1; AltName: Full=Smu-1 suppressor of mec-8 and unc-52 protein homolog Length=513	53/111 (48%),	3e-20	Q7ZVA0.1	GI:82241387	GO:0005737, GO:0005634,	*genolin_c15411 326 nt, Length = 3176, Expect = 1e-098,Identities = 204/211 (96%)*
131	scaffold98_Gene90_811430_811661	No hit						*gb|JG192367.1|JG192367 LUSME1NG-RP-035_D09_21FEB2007_073 LUSME1NG Linum usitatissimum, cDNA, mRNA sequence, Length = 647,Expect = e-128, Identities = 232/232 (100%)*
132	scaffold98_Gene91_816596_818534	No hit						*genolin_c25312 373 nt, Length = 372, Expect = 1e-081,Identities = 155/155 (100%)*
133	scaffold98_Gene92_823055_827063	Full=Pentatricopeptide repeat-containing protein At1g30610, chloroplastic; AltName: Full=Protein EMBRYO DEFECTIVE 2279; Flags: Precursor Length=1006	179/413 (43%),	3e-136	Q9SA76.1	GI:75200328	GO:0009507, GO:0009793,	*gb|EB712625.1|EB712625 LuP12026D03R LuP12 Linum usitatissimum cDNA clone LuP12026D03, mRNA, sequence Length = 399,Expect = e-129,Identities = 238/239 (99%)*

134	scaffold98_Gene93_837426_839430	Full=Coatomer subunit epsilon-1; AltName: Full=Epsilon-coat protein 1; Short=Epsilon-COP 1; AltName: Full=Epsilon1-COP Length=287	83/127 (65%)	4e-37	Q9MAX6.1	GI:75336169	GO:0030126, GO:0005198,GO:0015031, GO:0006890,	*gb|JG214482.1|JG214482 LUSPS1AD_RP_106_A17_14AUG2008_079 LUSPS1AD Linum usitatissimum cDNA, mRNA sequence, Length = 931, Expect = 3e-088,Identities = 166/166 (100%)*
135	scaffold98_Gene94_839858_840595	Full=Small nuclear ribonucleoprotein-associated protein B; Short=snRNP-B; AltName: Full=Sm protein B; Short=Sm-B; Short=SmB Length=199	56/91 (62%)	4e-32	Q05856.1	GI:10720262	GO:0015030, GO:0071013, GO:0045495,GO:0071011, GO:0030532, GO:0003723, GO:0007281, GO:0008406,GO:0007052, GO:0000398,	*gb|JG223194.1|JG223194 LUSST4AD-T3-021_O14_15SEP2009_049 LUSST1AD Linum usitatissimum, cDNA, mRNA sequence Length = 927, Expect = 0.0, Identities = 639/642 (99%), Gaps = 1/642 (0%)*
136	scaffold98_Gene95_841201_847125	Full=60S ribosomal protein L19-3 Length=208	99/105 (94%)	1e-45	P49693.3	GI:19924280	GO:0022625,GO:0005886, GO:0003735, GO:0006412,	*LUSEN1NG_RP_185_D12_28MAR2007_090.ab1, Length = 717,Expect = 0.0, Identities = 472/495 (95%), Gaps = 2/495 (0%)*
137	scaffold98_Gene96_849561_850950	Full=60S ribosomal protein L19-3 Length=208	99/105 (94%)	3e-48	P49693.3	GI:19924280	GO:0022625,GO:0005886, GO:0003735, GO:0006412	*gb|JG217339.1|JG217339 LUSPS1AD_RP_115_C24_18AUG2008_094 LUSPS1AD Linum usitatissimum,cDNA, mRNA sequence Length = 804, Expect = e-178,Identities = 316/316 (100%)*
138	scaffold98_Gene97_854177_858345	Full=Subtilisin-like protease; AltName: Full=Cucumisin-like serine protease; Flags: Precursor Length=757	50/139 (36%),	2e-16	O65351.1	GI:75099392	GO:0048046,GO:0009505, GO:0004252, GO:0080001, GO:0048359, GO:0043086, GO:0006508,	*gb|JG181098.1|JG181098 LUSLE4AD-T3-051_H19_13NOV2009_074 LUSLE1AD Linum usitatissimum cDNA, mRNA sequence Length = 687, Expect = 0.0,Identities = 441/453 (97%)*
139	scaffold98_Gene98_866879_867655	No hit						*Database: N:\Warehouse-Cloutier\Raja\flax-ESTs-NCBI-TUFGEN-FRENCH\All_flax-ESTs-NCBI-TUFGEN-FRENCH.txt, 462,190 sequences; 259,282,616 total letters,***** No hits found *******

Legend:

(green)	BLAST x hit vs UniProtKB (No Hits)
(blue)	BLASTx hit vs UniProtKB (less than 34 aminoacids or 35% similarity)
(red)	BLASTn hit against Flax-ESTs (No Hits)
(yellow)	BLASTn hit against Flax-ESTs (less than 80 bp or 80% similarity)
