# Supplementary material for: Genetic characterization of a core collection of flax (Linum usitatissimum L.) suitable for association mapping studies and evidence of divergent selection between fiber and linseed types
Source: BMC Plant Biol. 2013 May 6;13:78. doi: 10.1186/1471-2229-13-78 (PMC3656786; doi:10.1186/1471-2229-13-78)
Supplement: Additional file 5: Figure S3 — (Portable Document Format file) GO-slim annotations of gene products predicted from nine non-neutral candidate genomic regions between fiber flax and linseed groups. (a) Molecular function. (b) Biological process. (c) Cellular component. [file 1471-2229-13-78-S5.pdf]

(a)

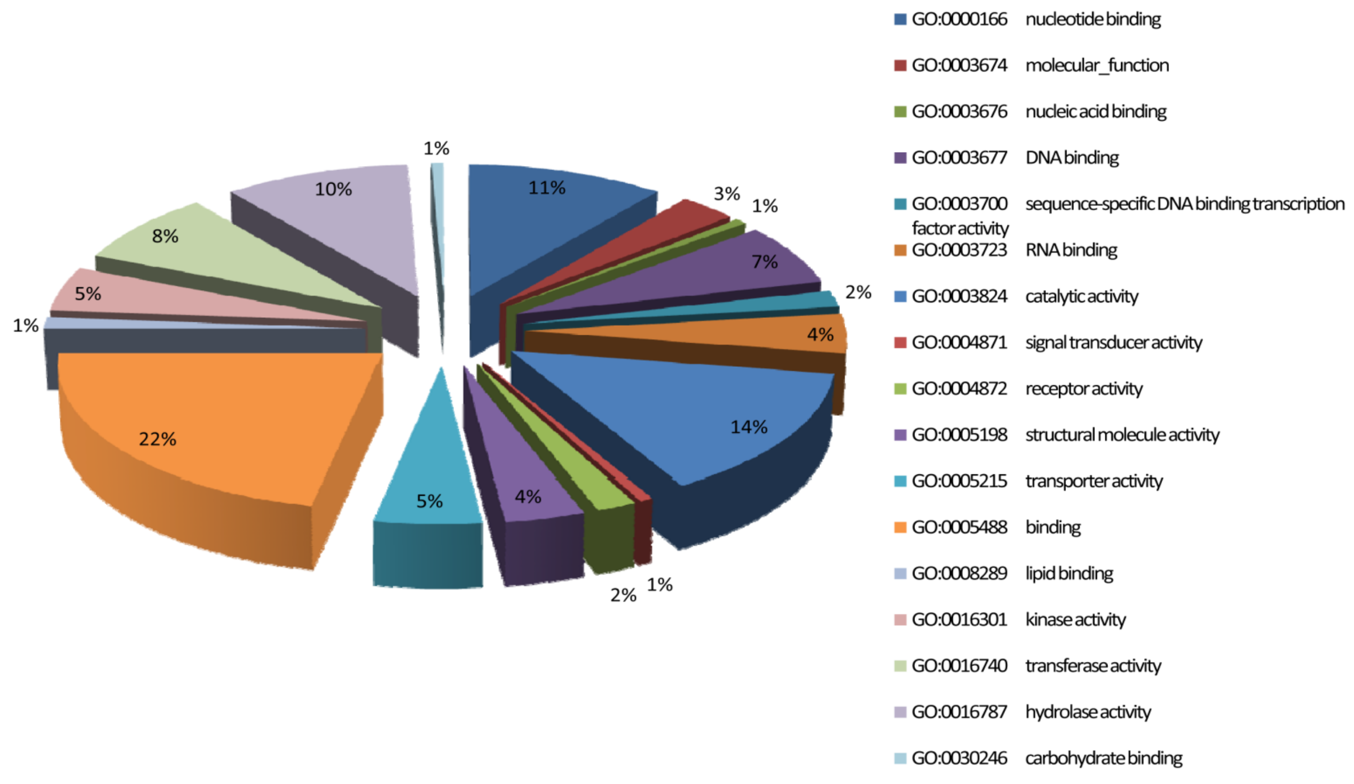
1%
11%
3%
1%
7%
2%
4%
14%
1%
2%
4%
5%
22%
1%
5%
8%
10%
GO:0000166 nucleotide binding
GO:0003674 molecular_function
GO:0003676 nucleic acid binding
GO:0003677 DNA binding
GO:0003700 sequence-specific DNA binding transcription factor activity
GO:0003723 RNA binding
GO:0003824 catalytic activity
GO:0004871 signal transducer activity
GO:0004872 receptor activity
GO:0005198 structural molecule activity
GO:0005215 transporter activity
GO:0005488 binding
GO:0008289 lipid binding
GO:0016301 kinase activity
GO:0016740 transferase activity
GO:0016787 hydrolase activity
GO:0030246 carbohydrate binding

(b)

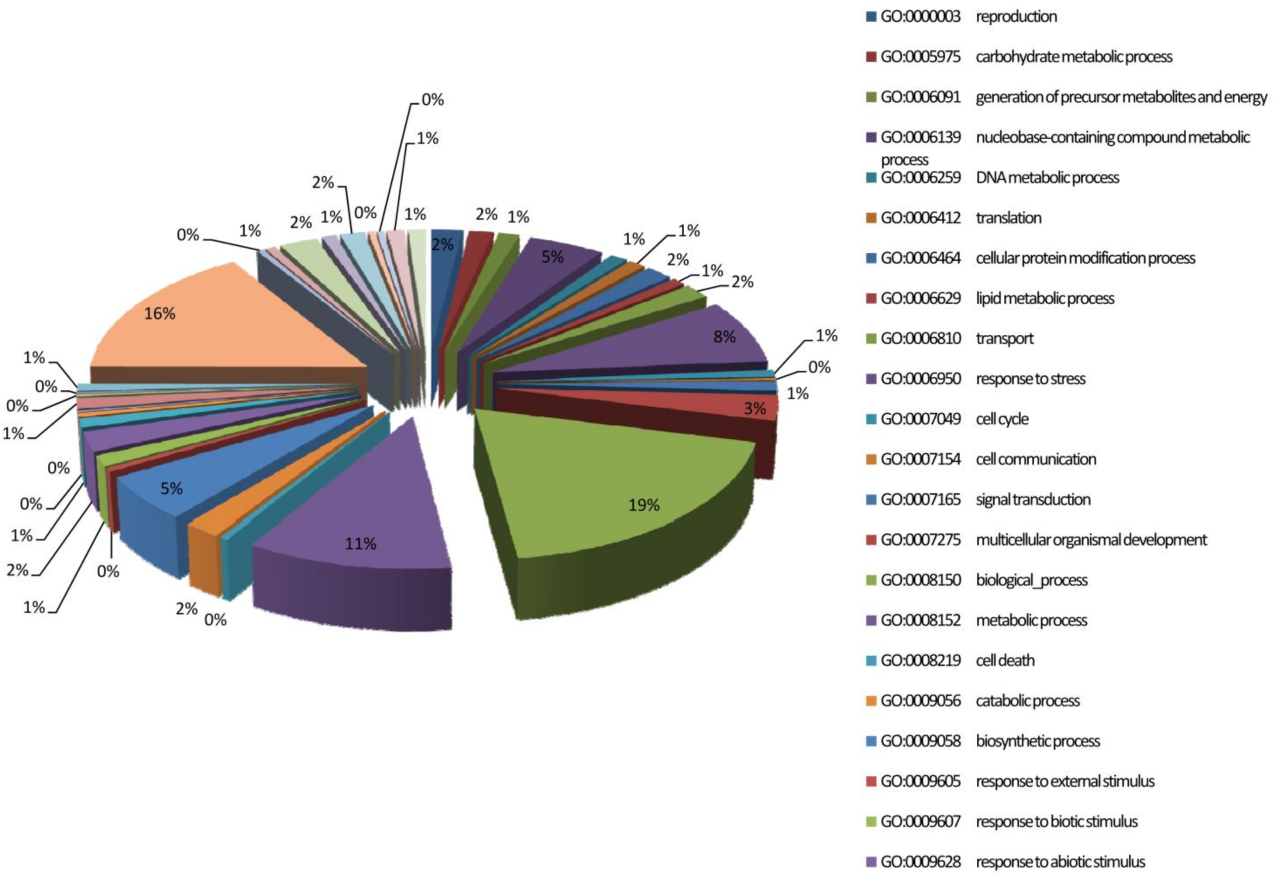
GO:0000003 reproduction
GO:0005975 carbohydrate metabolic process
GO:0006091 generation of precursor metabolites and energy
GO:0006139 nucleobase-containing compound metabolic process
GO:0006259 DNA metabolic process
GO:0006412 translation
GO:0006464 cellular protein modification process
GO:0006629 lipid metabolic process
GO:0006810 transport
GO:0006950 response to stress
GO:0007049 cell cycle
GO:0007154 cell communication
GO:0007165 signal transduction
GO:0007275 multicellular organismal development
GO:0008150 biological_process
GO:0008152 metabolic process
GO:0008219 cell death
GO:0009056 catabolic process
GO:0009058 biosynthetic process
GO:0009605 response to external stimulus
GO:0009607 response to biotic stimulus
GO:0009628 response to abiotic stimulus

(c)

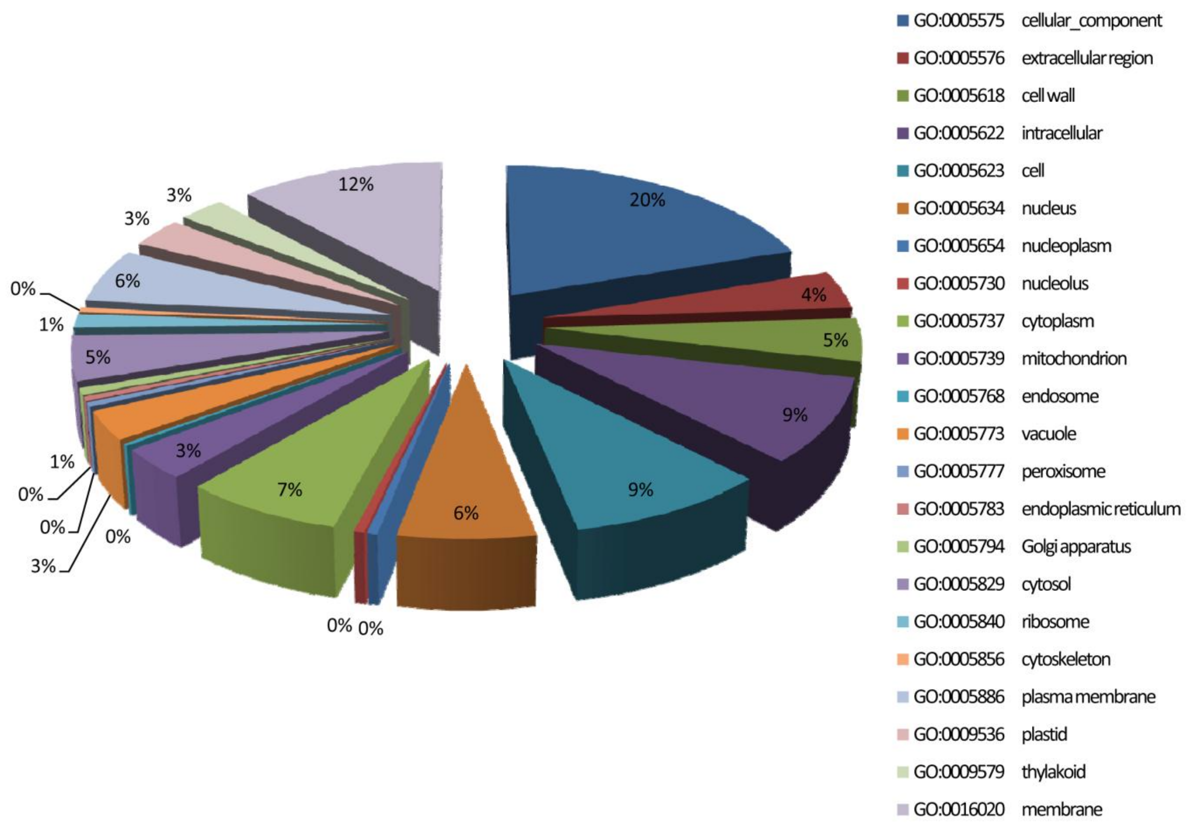

Figure S3. GO-slim annotations of gene products predicted from nine non-neutral candidate genomic regions between fiber flax and linseed groups. **(a)** Molecular function. **(b)** Biological process. **(c)** Cellular component.
