# Supplementary material for: Genetic characterization of a core collection of flax (Linum usitatissimum L.) suitable for association mapping studies and evidence of divergent selection between fiber and linseed types
Source: BMC Plant Biol. 2013 May 6;13:78. doi: 10.1186/1471-2229-13-78 (PMC3656786; doi:10.1186/1471-2229-13-78)
Supplement: Additional file 6: Table S3 — (Portable Document Format file) Core collection data including accession number, accession name, origin and improvement status. CN = Canadian number, Plant Gene Resources of Canada (PGRC). [file 1471-2229-13-78-S6.pdf]

Table S3 Core collection data including accession number, accession name, origin and imporvement stat

Sample	Accession number	Name	Type	Improvement status	Origin
1	CN18973	AC WATSON	Oil	Cultivar	CAN
2	CN18979	FLANDERS	Oil	Cultivar	CAN
3	CN18980	SOMME	Oil	Cultivar	CAN
4	CN18981	CDC VALOUR	Oil	Cultivar	CAN
5	CN18982	EVELIN	Fiber	Cultivar	FRA
6	CN18983	LAURA	Fiber	Cultivar	NLD
7	CN18986	HERMES	Fiber	Cultivar	FRA
8	CN18987	VIKING	Fiber	Cultivar	NLD
9	CN18988	ARIANE	Fiber	Cultivar	FRA
10	CN18989	ATALANTE	Oil	Cultivar	FRA
11	CN18991	NIKE	Fiber	Cultivar	POL
12	CN18993	LINDA	Oil	Cultivar	NLD
13	CN18994	VERNE	Oil	Cultivar	USA
14	CN18997	RAISA	Fiber	Cultivar	NLD
15	CN18998	ESCALINA	Fiber	Cultivar	NLD
16	CN19001	MARINA	Fiber	Cultivar	NLD
17	CN19003	AC MCDUFF	Oil	Cultivar	CAN
18	CN19004	AC EMERSON	Oil	Cultivar	CAN
19	CN19005	AC LINORA	Oil	Cultivar	CAN
20	CN19007	LIN-1724	Oil	Breeding material	ETH
21	CN19017	CDC NORMANDY	Oil	Cultivar	CAN
22	CN19157	OTTAWA 829-C	Oil	Cultivar	CAN
23	CN19158	OTTAWA 770B	Oil	Cultivar	CAN
24	CN19159	DIADEM	Oil	Cultivar	CAN
25	CN19160	BOLLEY GOLDEN	Oil	Cultivar	USA
26	CN30860	Kirovogradskij 71	Oil	Cultivar	UKR
27	CN30861	Kubanskij	Oil	Cultivar	UNK
28	CN32542	VNIIL-17	Fiber	Cultivar	RUS
29	CN32546	Korostenskij 3	Fiber	Cultivar	UKR
30	CN33385	LINOTT	Oil	Cultivar	CAN
31	CN33386	NORALTA	Oil	Cultivar	CAN
32	CN33388	REDWOOD 65	Oil	Cultivar	CAN
33	CN33389	ROCKET	Oil	Cultivar	CAN
34	CN33390	NATASJA	Fiber	Cultivar	NLD
35	CN33393	Domtar Selection	Fiber	Cultivar	UNK
36	CN33397	DUFFERIN	Oil	Cultivar	CAN
37	CN33399	BISON	Oil	Cultivar	USA
38	CN33400	NORSTAR	Oil	Cultivar	USA
39	CN33992	CULBERT	Oil	Cultivar	USA
40	CN35791	TVERCA	Fiber	Cultivar	RUS
41	CN37286	MCGREGOR	Oil	Cultivar	CAN
42	CN40081	NATASJA	Fiber	Cultivar	NLD
43	CN52732	NORLIN	Oil	Cultivar	CAN
44	CN96845	CIli-642	Oil	Cultivar	RUS
45	CN96846	CIli-643	Oil	Cultivar	RUS
46	CN96911	CIli-1407	Oil	Cultivar	TUR
47	CN96958	CIli-1455	Oil	Landrace	TUR
48	CN96962	CIli-1458	Oil	Cultivar	TUR
49	CN96974	CIli-1470	Oil	Landrace	IND
50	CN96988	CIli-1499	Oil	Cultivar	ETH

Table S3 continued					
51	CN96991	CIli-1502	Oil	Cultivar	ETH
52	CN96992	CIli-1503	Oil	Cultivar	ETH
53	CN97004	CIli-1519	Oil	Cultivar	ETH
54	CN97050	CIli-1924	Oil	Cultivar	IRN
55	CN97056	CIli-1930	Oil	Cultivar	PAK
56	CN97064	CIli-1938	Oil	Cultivar	PAK
57	CN97072	CIli-1946	Oil	Landrace	PAK
58	CN97083	CIli-1957	Oil	Landrace	PAK
59	CN97092	CIli-1991	Oil	Cultivar	PAK
60	CN97096	CIli-1995	Oil	Cultivar	PAK
61	CN97103	CIli-2002	Oil	Cultivar	PAK
62	CN97129	CIli-2028	Oil	Landrace	IRN
63	CN97129B	CIli-2028B	Oil	Landrace	IRN
64	CN97139	CIli-2038	Oil	Cultivar	IRN
65	CN97147	CIli-2046	Oil	Cultivar	TUR
66	CN97153	CIli-2052	Oil	Cultivar	TUR
67	CN97176	HORAL	Oil	Cultivar	CZE
68	CN97180	Sorth Behbehan	Fiber	Cultivar	IRN
69	CN97214	CIli-2295	Oil	Cultivar	ARG
70	CN97238	No. 1048	Oil	Cultivar	HUN
71	CN97287	Lina Deta	Oil	Cultivar	HUN
72	CN97300	RAJA	Oil	Cultivar	HUN
73	CN97306	N.P. (R.R.) 9	Oil	Cultivar	IND
74	CN97307	N.P. (R.R.) 37	Oil	Cultivar	IND
75	CN97308	N.P. (R.R.) 38	Oil	Cultivar	IND
76	CN97312	T. 126	Oil	Cultivar	IND
77	CN97321	CIli-2528	Oil	Cultivar	ROM
78	CN97334	MOCORETA	Oil	Cultivar	ARG
79	CN97341	H723 F3-6-3-3-4-2-2	Unknown	Cultivar	ARG
80	CN97350	de metcha 1-3-3 Vilm	Oil	Cultivar	FRA
81	CN97351	de metcha 1-3-6 Vilm	Fiber	Cultivar	FRA
82	CN97366	Texas S. 4-6 Walsh x New Golden	Oil	Cultivar	USA
83	CN97377	Reserve (N. Dak. Res. 155)	Oil	Cultivar	USA
84	CN97392	NOVELTY	Oil	Cultivar	CAN
85	CN97393	Sel. C.I. 21-2 Jalaun	Oil	Cultivar	USA
86	CN97396	Res. x Hoshangabad (C.I. 19 x C.I. 140)	Oil	Cultivar	USA
87	CN97397	Sel. C.I. 19-47 Pale Blue	Unknown	Cultivar	USA
88	CN97402	No. Dak. No. 40,013	Unknown	Breeding material	USA
89	CN97403	LINOTA	Oil	Cultivar	USA
90	CN97404	Buda Sel.	Oil	Breeding material	USA
91	CN97404B	Buda Sel.B	Oil	Breeding material	USA
92	CN97406	No.Dak. Res. No. 52	Unknown	Breeding material	USA
93	CN97407	Rio (Long 79)	Oil	Cultivar	USA
94	CN97424	Tammes #3 White Involute	Fiber	Cultivar	NLD
95	CN97430	N.D. Nur. No. 1740 (G.36 a/21)	Oil	Breeding material	DEU
96	CN97430B	N.D. Nur. No. 1740 (G.36 a/21)B	Oil	Breeding material	DEU
97	CN97444	N.D. Resistant 714	Oil	Cultivar	USA
98	CN97452	Sel. of Minn. 281	Unknown	Cultivar	USA
99	CN97453	Pale Blue Sel. from N.D.R. 52	Unknown	Cultivar	USA
100	CN97458	CIli-469	Oil	Cultivar	NLD

Table S3 continued					
101	CN97463	Sel. of N.D.R. 114	Oil	Cultivar	USA
102	CN97470	Sagino	Oil	Cultivar	JPN
103	CN97475	Common White	Oil	Cultivar	RUS
104	CN97483	CIli-522	Unknown	Landrace	RUS
105	CN97484	CIli-523	Oil	Cultivar	RUS
106	CN97487	CIli-526	Oil	Cultivar	RUS
107	CN97489	CIli-531	Oil	Cultivar	RUS
108	CN97503	CIli-556	Fiber	Landrace	RUS
109	CN97520	CIli-576	Oil	Cultivar	RUS
110	CN97529	CIli-589	Oil	Cultivar	RUS
111	CN97530	CIli-590	Fiber	Landrace	RUS
112	CN97531	CIli-593	Fiber	Landrace	RUS
113	CN97533	CIli-595	Fiber	Landrace	RUS
114	CN97571	Cyprus	Oil	Cultivar	CAN
115	CN97584	Minn. Sel. Winona x 770B F5	Unknown	Breeding material	USA
116	CN97584B	Minn. Sel. Winona x 770B F6-B	Unknown	Breeding material	USA
117	CN97586	Long 66 (non-ciliate)	Oil	Cultivar	USA
118	CN97587	Capa (Argentine)	Oil	Cultivar	USA
119	CN97604	CIli-758	Oil	Cultivar	RUS
120	CN97605	CIli-759	Oil	Landrace	RUS
121	CN97610	Tammes Type 2	Fiber	Cultivar	NLD
122	CN97613	Tammes Type 5	Oil	Cultivar	NLD
123	CN97616	Tammes Type 12	Fiber	Cultivar	NLD
124	CN97633	Royal	Oil	Cultivar	CAN
125	CN97639	CIli-835	Oil	Cultivar	USA
126	CN97639	CIli-835B	Oil	Cultivar	USA
127	CN97642	Renew	Oil	Cultivar	USA
128	CN97649	CIli-847	Oil	Cultivar	USA
129	CN97665	CIli-854	Fiber	Breeding material	USA
130	CN97670	No. 5242 - 1937	Unknown	Breeding material	USA
131	CN97671	J.W.S.	Oil	Cultivar	CAN
132	CN97679	CIli-897	Oil	Breeding material	USA
133	CN97679B	CIli-897B	Oil	Breeding material	USA
134	CN97689	CIli-908	Oil	Cultivar	USA
135	CN97718	CIli-946	Oil	Cultivar	USA
136	CN97728	CIli-956	Oil	Cultivar	USA
137	CN97740	Redson	Oil	Cultivar	USA
138	CN97749	Crystal	Oil	Cultivar	USA
139	CN97768	Mourisco, E730	Unknown	Cultivar	PRT
140	CN97871	Atlas (fiber)	Fiber	Cultivar	SWE
141	CN97873	Redwood (C.I. 980 x Redson)	Oil	Cultivar	USA
142	CN97881	Biwing x C.I. 980 (II-40-35)	Oil	Cultivar	USA
143	CN97886	Lusatia	Oil	Cultivar	DEU
144	CN97890	Maritime	Oil	Cultivar	USA
145	CN97907	Victory B	Oil	Cultivar	USA
146	CN97921	CIli-1185	Oil	Cultivar	USA
147	CN97953	10382/46	Oil	Cultivar	ARG
148	CN97958	10387/46	Oil	Cultivar	ARG
149	CN97961	10390/46	Oil	Cultivar	ARG
150	CN97967	10397/46	Oil	Cultivar	ARG

Table S3 continued					
151	CN97980	10410/46	Oil	Cultivar	ARG
152	CN98007	10442/46	Oil	Cultivar	ARG
153	CN98012	10447/46	Oil	Cultivar	ARG
154	CN98014	10451/46	Oil	Cultivar	ARG
155	CN98027	10469/46	Oil	Cultivar	ARG
156	CN98037	10479/46	Oil	Breeding material	ARG
157	CN98037B	10479/46B	Oil	Breeding material	ARG
158	CN98039	10481/46	Oil	Cultivar	ARG
159	CN98056	Hollandia	Oil	Cultivar	NLD
160	CN98056B	Hollandia-B	Oil	Cultivar	NLD
161	CN98057	CIli-1474	Oil	Cultivar	IND
162	CN98072	Unryu	Fiber	Cultivar	JPN
163	CN98100	Uruguay 36/48	Oil	Cultivar	URY
164	CN98109	CIli-1562	Oil	Cultivar	IND
165	CN98135	CIli-1596	Oil	Cultivar	IND
166	CN98150	Z 11637	Fiber	Unknown	NLD
167	CN98157	R.R. 38	Oil	Cultivar	IND
168	CN98165	1546-S	Oil	Cultivar	IRN
169	CN98176	1224-S	Oil	Cultivar	AFG
170	CN98192	CIli-1653	Oil	Cultivar	IRL
171	CN98193	L.G. 0189B	Unknown	Cultivar	MAR
172	CN98231	CIli-1749	Oil	Cultivar	USA
173	CN98237	CIli-1827	Oil	Cultivar	PAK
174	CN98239	CIli-1829	Oil	Cultivar	PAK
175	CN98240	CIli-1830	Oil	Landrace	IND
176	CN98240B	CIli-1830B	Oil	Landrace	IND
177	CN98242	CIli-1832	Oil	Landrace	IND
178	CN98250	CIli-1840	Oil	Cultivar	IND
179	CN98254	Basin	Oil	Cultivar	IND
180	CN98263	Chaurra Olajlen	Oil	Cultivar	HUN
181	CN98263	Chaurra Olajlen-B	Oil	Cultivar	HUN
182	CN98275	N 39/a La Plata	Oil	Cultivar	HUN
183	CN98276	N 39/b La Plata	Oil	Cultivar	HUN
184	CN98278	Karnobat 4	Oil	Cultivar	HUN
185	CN98279	Karnobat 5	Oil	Cultivar	ARG
186	CN98286	Mapun	Fiber	Cultivar	HUN
187	CN98303	Torok 11	Fiber	Cultivar	HUN
188	CN98363	N.P. 30	Oil	Cultivar	IND
189	CN98364	N.P. 31	Oil	Cultivar	IND
190	CN98370	N.P. 37	Oil	Cultivar	IND
191	CN98397	N.P. 65	Oil	Cultivar	IND
192	CN98398	N.P. 66	Oil	Cultivar	IND
193	CN98415	N.P. 86	Oil	Cultivar	IND
194	CN98440	N.P. 109	Oil	Cultivar	IND
195	CN98467	N.P. (RR.) 405	Oil	Cultivar	IND
196	CN98468	N.P. (RR.) 407	Oil	Cultivar	IND
197	CN98475	Flachskopf	Oil	Cultivar	DEU
198	CN98505	Varoneshski 1308	Oil	Cultivar	RUS
199	CN98535	Texas S. 32-1 Viking x Norsk	Oil	Cultivar	USA
200	CN98541	Texas S. 32-1 Viking x Norsk	Oil	Cultivar	USA

Table S3 continued					
201	CN98542	Amalla' H.D. Long Sel.	Oil	Cultivar	USA
202	CN98566	Rwd x Mar Minn. 61-2151	Oil	Breeding material	USA
203	CN98566B	Rwd x Mar Minn. 61-2151-B	Oil	Breeding material	USA
204	CN98566		Oil	Breeding material	USA
205	CN98569	CIli-2473	Oil	Unknown	IND
206	CN98610	Brawley R0001 (yel.sd. sel.)	Unknown	Cultivar	USA
207	CN98613	Br.B502 (Imp. x Punj. 473) 1008-2	Oil	Cultivar	USA
208	CN98634	Toba	Oil	Cultivar	ARG
209	CN98639	W5618RO-41	Oil	Cultivar	USA
210	CN98644	W5623RO-24	Unknown	Breeding material	USA
211	CN98683	Mapum M.A.	Oil	Cultivar	CZE
212	CN98689	Primus	Unknown	Cultivar	CZE
213	CN98704	Wicking Hegenan	Fiber	Cultivar	CZE
214	CN98708	Vitagold	Fiber	Cultivar	FRA
215	CN98710	Erythree	Fiber	Landrace	FRA
216	CN98712	Safi 1.4-2-1	Oil	Cultivar	FRA
217	CN98733	Bulgare a h	Oil	Breeding material	POL
218	CN98734	Karnobat 9	Oil	Cultivar	FRA
219	CN98741	Karnobat 1591 1.9	Oil	Breeding material	FRA
220	CN98742	Comun de Diaz	Unknown	Landrace	FRA
221	CN98752	Lina grosses graines Vilmorin No1	Oil	Cultivar	FRA
222	CN98753	Lina de Safi Vilmorin No2	Oil	Cultivar	FRA
223	CN98767	LG 0196	Oil	Cultivar	FRA
224	CN98773	Safi 1.1-2-5	Oil	Cultivar	FRA
225	CN98794	Lino de Cabiro	Oil	Cultivar	FRA
226	CN98806	CIli-2761	Oil	Breeding material	FRA
227	CN98807	CIli-2762	Oil	Cultivar	FRA
228	CN98812	Bison LN (67-I-46)	Oil	Cultivar	USA
229	CN98821	Foster ND14a (1605 x Minerva)	Oil	Cultivar	USA
230	CN98826	Common	Fiber	Cultivar	EGY
231	CN98829	Dolgunetz	Fiber	Cultivar	USA
232	CN98854	CIli-1573	Oil	Cultivar	HUN
233	CN98869	Field N. 17	Oil	Cultivar	TUR
234	CN98903	CIli-1753	Fiber	Breeding material	USA
235	CN98923	CIli-1774	Fiber	Breeding material	USA
236	CN98926	CIli-1777	Fiber	Breeding material	USA
237	CN98934	Wada Fiber	Fiber	Cultivar	USA
238	CN98946	Talmune Fiber	Fiber	Cultivar	USA
239	CN98954	Cascade Fiber	Fiber	Cultivar	USA
240	CN98961	CIli-1846	Oil	Cultivar	IND
241	CN98969	N.P. 15	Oil	Cultivar	IND
242	CN98973	N.P. 117	Oil	Cultivar	IND
243	CN98974	N.P. 118	Oil	Cultivar	IND
244	CN98982	N.P. (RR.) 272	Oil	Cultivar	IND
245	CN98984	Bonnydoon-9 (H39-9)	Oil	Cultivar	AUS
246	CN100547	Redwing	Oil	Cultivar	UNK
247	CN100629	CIli-2971	Oil	Cultivar	PAK
248	CN100674	CIli-3026	Oil	Cultivated material	ROM
249	CN100678	CIli-3030	Unknown	Cultivated material	ROM
250	CN100770	CIli-3250	Oil	Breeding material	USA

Table S3 continued					
251	CN100785	VERNE 93 SDT8914	Oil	Breeding material	USA
252	CN100790	Ghari 3	Unknown	Cultivated material	PAK
253	CN100795	Tammes Pale Blue	Fiber	Cultivar	NLD
254	CN100797	SP 2271	Unknown	Breeding material	NZL
255	CN100797B	SP 2271-B	Unknown	Breeding material	NZL
256	CN100799	N.P. 84	Unknown	Cultivated material	IND
257	CN100805	Floribus Roseis	Oil	Cultivar	CZE
258	CN100807	LIN-1062	Oil	?	AFG
259	CN100827	Safedak	Oil	Cultivar	SUN
260	CN100828	Winterlein	Unknown	Unknown	TUR
261	CN100837	LIN-1193	Oil	Unknown	TUR
262	CN100838	LIN-706	Oil	Unknown	CYP
263	CN100841	LIN-627	Oil	Unknown	UNK
264	CN100848	Ottawa 2152	Fiber	Cultivar	CAN
265	CN100851	Sumpersky Fa 13 Jenny	Oil	Cultivar	CZE
266	CN100852	Grandal	Unknown	Cultivar	PRT
267	CN100863	LIN-771	Oil	Breeding material	FRA
268	CN100864	Bjelo Katjacs	Fiber	Cultivar	HUN
269	CN100881	Deutscher Ollein	Oil	Cultivar	DEU
270	CN100883	Beta 201	Oil	Cultivar	HUN
271	CN100884	g. 12 Ruzokvety	Oil	Cultivar	CSK
272	CN100885	aus Lathyrus	Unknown	Unknown	GRC
273	CN100895	Karbin (landrace)	Unknown	Landrace	ETH
274	CN100910	Grandal (landrace)	Oil	Landrace	PRT
275	CN100928	Ocean	Oil	Cultivar	FRA
276	CN100929	Belinka	Fiber	Cultivar	NLD
277	CN100939	VNIIL-7939	Oil	Cultivar	RUS
278	CN100952	VIR-1270	Fiber	Unknown	AFG
279	CN101016	Zheltosemyannyi	Unknown	Cultivar	CHN
280	CN101026	6 V27-2 (pop.varieta)	Oil	Breeding material	MAR
281	CN101038	Nika	Fiber	Cultivar	BLR
282	CN101039	VNIIL-4767	Fiber	Breeding material	RUS
283	CN101052	L-93-2	Fiber	Breeding material	CHN
284	CN101053	L-8709-5-10	Fiber	Breeding material	CHN
285	CN101055	L-140-16	Fiber	Breeding material	RUS
286	CN101094	Torzhokskij 4	Fiber	Cultivar	RUS
287	CN101096	Novotorzhskij	Fiber	Cultivar	RUS
288	CN101099	Aleksim	Fiber	Cultivar	RUS
289	CN101114	VNIIL-5320	Fiber	Breeding material	RUS
290	CN101115	VNIIL-5321	Fiber	Breeding material	RUS
291	CN101116	VNIIL-5316	Fiber	Breeding material	RUS
292	CN101118	VNIIL-5316	Fiber	Breeding material	LTU
293	CN101119	VNIIL-3177	Fiber	Breeding material	RUS
294	CN101127	VNIIL-5317	Fiber	Breeding material	RUS
295	CN101132	VNIIL-5623	Oil	Breeding material	RUS
296	CN101136	Verchnevolzhkij	Fiber	Cultivar	RUS
297	CN101137	VNIIL-5613	Oil	Breeding material	RUS
298	CN101154	Belochka	Fiber	Cultivar	RUS
299	CN101208	VNIIL-5545	Oil	Cultivar	IND
300	CN101230	VNIIL-5520	Fiber	Breeding material	CHN

Table S3 continued					
301	CN101237	Artemida	Oil	Cultivar	LTU
302	CN101240	VNIIL-6182	Oil	Breeding material	LTU
303	CN101241	VNIIL-5325	Oil	Breeding material	RUS
304	CN101265	Amason	Oil	Cultivar	GBR
305	CN101279	VNIIL-5606	Oil	Breeding material	RUS
306	CN101286	Dakota Line 8	Oil	Breeding material	USA
307	CN101289	VNIIL-5680	Oil	Breeding material	RUS
308	CN101296	L. 270-68	Oil	Breeding material	RUS
309	CN101298	L. 541-02	Oil	Breeding material	RUS
310	CN101299	L. 00-207	Oil	Breeding material	RUS
311	CN101301	L. 1200-4-3	Oil	Breeding material	RUS
312	CN101307	LM-95	Oil	Breeding material	RUS
313	CN101308	VNIIL-180	Oil	Unknown	IND
314	CN101310	VNIIL-571	Oil	Unknown	IND
315	CN101325	VNIIL-1104	Oil	Unknown	GRC
316	CN101327	VNIIL-6148	Unknown	Unknown	ESP
317	CN101329	VNIIL-519	Oil	Unknown	EGY
318	CN101331	VNIIL-918	Oil	Unknown	TUR
319	CN101332	VNIIL-1046	Oil	Unknown	TUR
320	CN101338	VNIIL-655	Oil	Unknown	AFG
321	CN101348	VNIIL-742	Fiber	Unknown	RUS
322	CN101364	VNIIL-776	Fiber	Unknown	RUS
323	CN101366	VNIIL-725	Oil	Unknown	GEO
324	CN101367	VNIIL-2785	Oil	Unknown	GEO
325	CN101373	VNIIL-868	Oil	Unknown	ARM
326	CN101375	VNIIL-3531	Oil	Unknown	RUS
327	CN101378	VNIIL-409	Fiber	Unknown	UKR
328	CN101379	VNIIL-492	Fiber	Unknown	UKR
329	CN101382	Keteni Tekirdak Hagrobobu	Fiber	Unknown	TUR
330	CN101385	TR 35141	Fiber	Unknown	TUR
331	CN101386	TR 42713	Fiber	Unknown	TUR
332	CN101392	Tajga	Fiber	Cultivar	FRA
333	CN101394	Line 548-01	Fiber	Unknown	RUS
334	CN101395	Line 629-01	Fiber	Unknown	RUS
335	CN101396	Line 657-01	Fiber	Unknown	RUS
336	CN101397	Pskovski 2976	Fiber	Unknown	UKR
337	CN101401	G 2063-5-10	Fiber	Unknown	RUS
338	CN101402	VNIIL-5631	Fiber	Unknown	RUS
339	CN101403	L-500004-2-84	Fiber	Unknown	ROM
340	CN101404	L-60016-3-87	Fiber	Unknown	ROM
341	CN101405	Mures	Fiber	Unknown	ROM
342	CN101406	L-41	Fiber	Unknown	RUS
343	CN101407	Concurent	Fiber	Unknown	NLD
344	CN101413	Vimy	Oil	Cultivar	CAN
345	CN101416	China 1	Fiber	Breeding material	CHN
346	CN101417	China 2	Fiber	Breeding material	CHN
347	CN101419	China 4	Fiber	Breeding material	CHN
348	CN101421	China 5	Fiber	Breeding material	CHN
349	CN101448	Sel Clli -332 (C5)	Oil	Breeding material	CAN
350	CN101451	Sel Clli -400 (C5)	Unknown	Breeding material	CAN

Table S3 continued					
351	CN101454	Sel. of CIli-684 (C4)	Oil	Breeding material	CAN
352	CN101461	Sel. of CIli-1020 (C4)	Oil	Breeding material	CAN
353	CN101463	Sel. of CIli-1220 (C4)	Oil	Breeding material	CAN
354	CN101466	Sel. of CIli-1472 (C4)	Oil	Breeding material	CAN
355	CN101469	Sel. of CIli-1484 (C4)	Oil	Breeding material	CAN
356	CN101471	Sel CIli -1490 (C4)	Oil	Breeding material	CAN
357	CN101472	Sel. of CIli-1493 (C4)	Oil	Breeding material	CAN
358	CN101482	Sel. of CIli-1676 (C4)	Oil	Breeding material	CAN
359	CN101486	Sel CIli -1761 (short)	Fiber	Breeding material	CAN
360	CN101493	Sel CIli -1819 (C4)	Oil	Breeding material	CAN
361	CN101496	Sel. of CIli-1856 (LS)	Oil	Breeding material	CAN
362	CN101510	Sel CIli -1966 (C4)	Oil	Breeding material	CAN
363	CN101511	Sel CIli -1967 (C5)	Oil	Breeding material	CAN
364	CN101535	Sel CIli -2085 (C2)	Oil	Breeding material	CAN
365	CN101536	Sel CIli -2085 (C4)	Oil	Breeding material	CAN
366	CN101539	Sel CIli -2155 (C4)	Oil	Breeding material	CAN
367	CN101542	Sel CIli -2197 (C4)	Oil	Breeding material	CAN
368	CN101554	Sel CIli -2225 (C4)	Oil	Breeding material	CAN
369	CN101559	Sel CIli -2225 (C4)	Fiber	Breeding material	CAN
370	CN101560	Sel. of CIli-2289 (C2)	Oil	Breeding material	CAN
371	CN101565	Sel CIli -2410 (C6)	Oil	Breeding material	CAN
372	CN101572	Sel. of CIli-2560 (C4)	Unknown	Breeding material	CAN
373	CN101580	Sel. of CIli-2611 (C4)	Oil	Breeding material	CAN
374	CN101594	Sel. of CIli-2699 (C4)	Oil	Breeding material	CAN
375	CN101595	Sel CIli -2703 (C6/C4)	Oil	Breeding material	CAN
376	CN101596	Sel CIli -2719 (C4)	Oil	Breeding material	CAN
377	CN101598	Sel. of CIli-2734 (C4)	Oil	Breeding material	CAN
378	CN101600	Sel CIli -2748 (C4)	Oil	Breeding material	CAN
379	CN101610	Sel VIR-2404	Unknown	Breeding material	CAN
380	Linola989	Linola989	Oil	Cultivar	CAN
381	CDCGold	CDCGold	Oil	Cultivar	CAN
382	Macbeth	Macbeth	Oil	Cultivar	CAN
383	Shape	Shape	Oil	Cultivar	CAN
384	CDCSorrel	CDCSorrel	Oil	Cultivar	CAN
385	Atlas	Atlas	Fiber	Cultivar	SWE
386	CDCBethune	CDCBethune	Oil	Cultivar	CAN
387	CDCMons	CDCMons	Oil	Cultivar	CAN
388	CrepitamTabor	CrepitamTabor	Fiber	Cultivar	CAN
389	DoubleLow	DoubleLow	Oil	Cultivar	CAN
390	E1747	E1747	Oil	Cultivar	CAN
391	FP2214	FP2214	Oil	Cultivar	CAN
392	FP2270	FP2270	Oil	Cultivar	CAN
393	G1186-94	G1186-94	Fiber	Cultivar	CAN
394	Hanley	Hanley	Oil	Cultivar	CAN
395	Lirina	Lirina	Oil	Cultivar	CAN
396	M5791	M5791	Oil	Cultivar	CAN
397	M96006	M96006	Oil	Cultivar	CAN
398	PrairieBlue	PrairieBlue	Oil	Cultivar	CAN
399	PrairieGrande	PrairieGrande	Oil	Cultivar	CAN
400	PrairieThunder	PrairieThunder	Oil	Cultivar	CAN

Table S3 continued					
401	SP2047	SP2047	Oil	Cultivar	CAN
402	S95407	S95407	Oil	Cultivar	CAN
403	UGG102-2	UGG102-2	Oil	Cultivar	CAN
404	UGG146-1	UGG146-1	Oil	Cultivar	CAN
405	UGG5-5	UGG5-5	Oil	Cultivar	CAN
406	Viking(European)	Viking(European)	Fiber	Cultivar	EU
407	YSED18	YSED18	Oil	Cultivar	CAN

CN = Canadian number, Plant Gene Resources of Canada (PGRC)

us
