# Supplementary material for: Genetic characterization of a core collection of flax (Linum usitatissimum L.) suitable for association mapping studies and evidence of divergent selection between fiber and linseed types
Source: BMC Plant Biol. 2013 May 6;13:78. doi: 10.1186/1471-2229-13-78 (PMC3656786; doi:10.1186/1471-2229-13-78)
Supplement: Additional file 7: Figure S4 — (Portable Document Format file) Distribution of the 407 flax accessions of the core collection. (a) geographical origin. (b) improvement status. [file 1471-2229-13-78-S7.pdf]

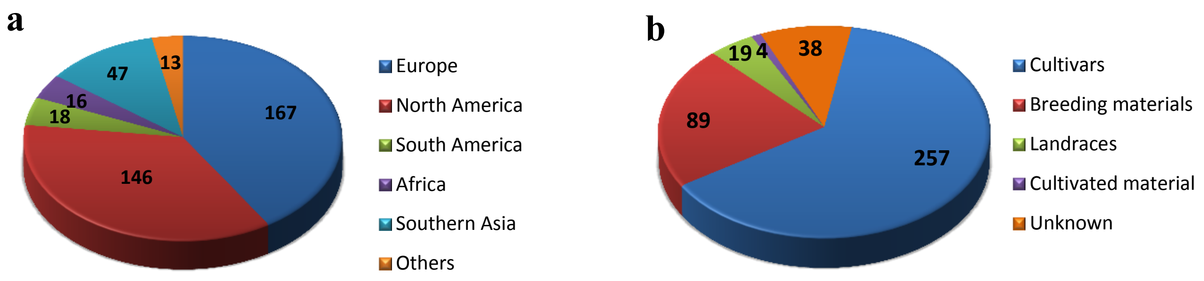

Figure S4. Distribution of the 407 flax accessions of the core collection. **(a)** geographical origin. **(b)** improvement status.
